# Can care coordination across levels be improved through the implementation of participatory action research interventions? Outcomes and conditions for sustaining changes in five Latin American countries

**DOI:** 10.1186/s12913-020-05781-7

**Published:** 2020-10-12

**Authors:** Ingrid Vargas, Pamela Eguiguren, Amparo-Susana Mogollón-Pérez, Isabella Samico, Fernando Bertolotto, Julieta López-Vázquez, María-Luisa Vázquez

**Affiliations:** 1 Health Policy and Health Services Research Group, Health Policy Research Unit, Consortium for Health Care and Social Services of Catalonia, Avenida Tibidabo 21, 08022 Barcelona, Spain; 2 Escuela de Salud Pública Dr. Salvador Allende Gossens, Facultad de Medicina, Universidad de Chile, Avenida Independencia, 939 Santiago de Chile, Chile; 3 Escuela de Medicina y Ciencias de la Salud, Universidad del Rosario, Cra 24 No. 63C-69, Quinta Mutis, 11001 Bogotá, Colombia; 4 Grupo de Estudos de Gestão e Avaliação em Saúde, Instituto de Medicina Integral Prof. Fernando Figueira, Rua Dos Coelhos No. 300, Boa Vista, 50070-550 Recife Brazil; 5 Facultad de Enfermería, Universidad de la República, Avenida 18 de Julio 124, 11200 Montevideo, Uruguay; 6 Instituto de Salud Pública, Universidad Veracruzana, Avenida Dr. Luis Castelazo Ayala s/n. Col. Industrial Ánimas, 91190 Xalapa, Veracruz, Mexico

**Keywords:** Care coordination, Care integration, Integrated delivery systems, Participatory action research, Health services research, Qualitative research, Implementation science, Outcomes assessment, Physicians, Latin America

## Abstract

**Background:**

Finding new strategies for care integration has become a policy priority for many fragmented health systems in Latin America. Although the implementation of interventions through a participatory action research (PAR) approach is considered to be more effective in achieving organizational change, its application is scarce.

This study, part of the research project Equity-LA II, aims to analyze the impact of PAR interventions on care coordination across levels, and key aspects for their sustainability and transferability, from the stakeholder viewpoint in healthcare networks of Brazil, Chile, Colombia, Mexico and Uruguay. Different interventions were designed and implemented through a PAR process to improve communication and clinical agreement between primary care and secondary care doctors: joint meetings to discuss clinical cases and/or training; shared care guidelines; offline virtual consultations; a referral and reply letter; and an induction program.

**Methods:**

A qualitative, descriptive-interpretative study was conducted in the healthcare network of each country. Focus groups and semi-structured individual interviews were conducted with a criterion sample of participants: local steering committee (29) and professional platform members (28), other health professionals (49) and managers (28). Thematic content analysis was conducted, segmented by country and type of intervention.

**Results:**

Informants highlighted that joint meetings based on reflexive methods contributed substantially to improving contextually relevant elements of clinical management coordination – communication in patient follow-up, clinical agreement, appropriateness of referrals – and also administrative coordination. The meetings, alongside the PAR process, also helped to improve interaction between professionals – knowing each other personally and mutual trust – thus fostering willingness to collaborate. The PAR approach, moreover, served to spread awareness of the coordination problems and need for intervention, encouraging greater commitment and interest in participating. No noteworthy contributions were identified in remaining interventions due to low uptake. A necessary condition for the sustainability and replicability was that PAR process had to be used appropriately in a favourable context.

**Conclusions:**

Evidence is provided on the substantial contribution of interventions to improving locally relevant clinical coordination elements and professional interaction when implemented through an adequate PAR process (in terms of time, method and participation levels), a necessary condition for their sustainability and replicability.

## Background

Finding new integrated care strategies that can lead to better outcomes has become a policy priority in many countries, especially in areas such as Latin America where healthcare systems are highly fragmented [1, 2]. In this kind of setting, constant causes for concern in terms of quality of care include limited communication and clinical data exchange between primary care (PC) and secondary care (SC) health professionals in the transition of patients between PC and SC levels, access barriers in referrals to SC and, to a lesser degree but still relevant, disagreement on treatments between PC and SC doctors, along with the duplication of diagnostic tests [3]. Measures to address these problems are generally based on the top-down implementation of care coordination interventions designed at national or regional level without the participation of healthcare professionals. Their adaptation and dissemination in the local context is limited, and they are usually based more on the standardization of clinical procedures than on direct feedback between professionals [4]. It is of no surprise, therefore, that studies conducted in the region show low levels of adoption of the coordination mechanisms introduced and point to the need to explore other ways of implementing changes in the health services that take local priorities into account and achieve better outcomes [4].

In this respect, in recent years participatory action research (PAR) has developed a growing reputation as an effective tool for achieving organizational change in health systems and closing the gap between theory and professional practice [5]. PAR is defined here as a systematic inquiry, with the collaboration of those affected by the issue being studied, for purposes of education and taking action or effecting social change [6]. Its effectiveness has been attributed to the characteristics inherent to PAR [7], which are also identified in the literature on organizational change [8–13] as important conditions for achieving planned change: the cyclical and reflexive process of research-action, the collaboration between local actors and the researchers who act as facilitators [14] and the method based on the egalitarian participation of all actors, shared decision-making and collective construction [15]. Involvement of the practitioners throughout the research process, including decision-making, firstly lends greater relevance and validity to interventions designed to resolve common practical problems in their working day, and secondly creates more interest and positive feedback regarding the changes being instituted, increasing their sustainability over time [14, 16].

Despite the potential benefits of the PAR approach, its application in healthcare organizations has so far mainly been concentrated within the field of hospital nursing and limited to certain countries such as UK, Australia and USA [5, 14, 17, 18]. It has been used very little in low- and middle-income countries [19] and in the implementation of interventions aimed at doctors [9] or at professionals of different healthcare levels in a network.

### A comprehensive conceptual framework for the analysis of care coordination

This study is based on a comprehensive conceptual framework on care coordination across care levels that addresses the different types and dimensions of clinical coordination [3, 20], the care coordination mechanisms [20] to improve them and the influencing factors [3]. Care coordination is defined as the harmonious connection of the different services needed to provide care to a patient throughout the care continuum in order to achieve a common objective without conflicts [21]. Most of the literature on care coordination refers to two interrelated types of care coordination: *information coordination*, or the transfer and use of the patient clinical information needed to coordinate activities between providers, and *clinical management coordination*, or the provision of care in a sequential and complementary way, which encompasses three dimensions - care coherence, follow-up and accessibility across levels of care [22]. However, there is also a third type: *administrative coordination*, or the coordination of patient access across the continuum of services according to their needs. This latter is less frequently conceptualized by scholars but equally important, especially in highly fragmented healthcare systems with significant barriers in access across care levels [23].

A wide range of strategies can be used to improve care coordination, from introducing a single care coordination mechanism (based on *standardization of processes/skills*, e.g. clinical guidelines, ongoing medical training or based on *feedback between professionals,* e.g. multidisciplinary teams, joint clinical case conferences, liaison roles or integrated information systems), to combining various different mechanisms in a more comprehensive approach [1]. There is no one ideal combination of mechanisms, but organizational theory suggests that the use of standardization-based mechanisms is appropriate in situations that can be anticipated and do not necessarily require a rapid response, and that feedback mechanisms are more suitable for highly uncertain situations, and/or those involving specialized and interdependent activities [24]. In this case, coordination is achieved by encouraging contact between individuals in order to solve the problem at the same level at which the information is generated [25]. Although further research is required on the intermediate and final outcomes of these strategies, evaluations of their effectiveness indicate, firstly, that introducing multidisciplinary teams and comprehensive programs such as case and disease management programs has a greater impact on health outcomes, and secondly, that their effects depend on contextual factors and on how well they are designed and implemented [1].

Studies on the contextual factors that influence the use of mechanisms highlight – in addition to organizational elements such as lack of time or resources to be able to use them [26] – the importance of factors related to individuals: professionals’ attitude towards collaborating with those of the other care level, knowing each other personally, mutual trust [3, 27–29] and motivation to use the mechanism [26]. It is precisely in enhancing these individual factors that the PAR approach shows most promise. Although it is rarely employed in the design and implementation of coordination across care levels [30], assessments of its application in other fields indicate relevant outcomes, including improved attitude-related factors, such as participants’ greater motivation and enthusiasm, and increased awareness of the problem [5, 14]; improved interaction between participants such as team work, group cohesion, and communication [5, 9, 14]; and the development of new skills and tools [5, 14]. However, few of these assessments analyze from the participants’ perspective the factors that influence the implementation of interventions and the conditions needed for the changes to be sustained over time, which are key aspects for their replicability in other contexts.

### The Equity-LA II project and the PAR process of designing and implementing interventions to improve clinical coordination

This study is part of a wider implementation research project, Equity-LA II [20], which aims to evaluate the effectiveness of interventions (designed and implemented through a PAR process) to improve clinical coordination across care levels in healthcare networks of Argentina, Brazil, Chile, Colombia, Mexico and Uruguay. It adopts a quasi-experimental design (controlled before-and-after) with one intervention and one control public healthcare network in each country [20], using a participatory and mixed-methods approach. The healthcare networks were selected according to the following criteria: a) provision of a continuum of health services including at least PC and SC; b) provision of care to a defined population; c) mainly covering urban areas of low or medium-low socioeconomic status; d) willingness to participate [20].

The Equity-LA II project combines quantitative methods to analyze the effectiveness of the interventions on care coordination [31] and continuity of care [32] with qualitative data collection methods to evaluate the design and implementation process [33] and the perceived contributions of the interventions to care coordination from the participants’ viewpoint. The latter objective is the focus of this paper.

The research was carried out in six Latin American middle-income countries, representing different types of healthcare systems [3]. However, all six health systems are segmented by population groups according to socioeconomic or employment status [34, 35], with a public subsystem and a private one. The public sector, which is the focus of this study, is financed by social security contributions and/or taxes and is generally aimed at the lower income population and/or those without social security. In all countries, the public healthcare subsystem is organized in networks of health services, and care is organised by levels of complexity, with PC as the entry point and coordinator of patient care and SC in a supporting role, requiring a referral from PC for access to the specialist [36].

The participatory action research (PAR) process in each country was led by a local steering committee (LSC) in the healthcare network selected to carry out the intervention. The LSC comprised managers from both care levels, health professionals in some cases, and the researchers in the role of facilitators. In the first phase, the baseline study, evidence was produced on cross-level care coordination and continuity in the networks [3, 4, 27, 37], which was then presented to and discussed with the professionals. In the second phase, an inter-level professional platform (PP) was created with those interested in taking action, through which the baseline study results were problematized and interventions were chosen [38]. Subsequently, the interventions were designed and implemented through three types of process: 1) in Colombia, Brazil and Mexico, two PARs cycles: i) a short initial design by the PP and/or LSC followed by implementation and ii) adjustment and implementation; 2) in Chile, long design with high participation through inter-level working groups of professionals and LSC, in several cycles of action-reflection, with pilot tests and implementation; and 3) in Argentina and Uruguay, open design to be agreed upon later with the PP and subsequent implementation [33]. Argentina is not included in this paper because the selected intervention (shared care guideline for hypertension and diabetes) was not implemented [38].

All interventions were aimed at improving communication and clinical agreement between PC and SC doctors and can be classified into three basic groups: i) joint meetings between PC and SC doctors to discuss clinical cases and/or for training purposes, in person in Brazil (mental health), Colombia (chronic diseases), and Mexico (maternal care and chronic diseases) and online in Chile. Other health professionals also took part (e.g. nurses, physiotherapists, psychologists, etc.), albeit to a greater extent in Chile; ii) offline virtual consultations between PC and SC doctors, via email in Brazil (mental health) and a digital platform in Mexico (chronic diseases); iii) other types of care coordination interventions: in Brazil, the design (in cross-level joint meetings) and implementation of shared care guidelines for diabetes; in Uruguay, a multi-component strategy to encourage the use of the referral and reply letter; and in Chile, an induction program to working in the healthcare network to promote a common identity and shared vision (Supplementary Table 1 “Characteristics of the implemented interventions” in Additional File 2).

Participation in the interventions was greater in those that lasted longest: joint meeting attendance in Colombia (16 months) and the use of diabetes shared care guidelines in Brazil (21 months). More PC doctors participated than SC doctors, except in the case of virtual joint clinical conferences in Chile (13 months), where the number of specialists that took part was also high. By contrast, participation figures were low for offline virtual consultations in Brazil (3 months) and Mexico (14 months), use of the referral and reply letter in Uruguay (7 months) and joint meetings on mental health in Brazil (7 months) [33]. An analysis of the PAR design and implementation process and the content of the interventions has already been published in two previous publications [33, 38].

The aim of this paper is to analyze the contribution of PAR interventions implemented in public healthcare networks to improving care coordination between PC and SC and its influencing factors, as well as conditions for the sustainability and the applicability of the interventions to other contexts, from the viewpoint of stakeholders in the above described health systems of Latin America.

## Methods

### Study design and study settings

A qualitative, descriptive-interpretative study was conducted to analyse, from the stakeholder perspective, the implementation of care coordination interventions through participatory action research, focusing on their results on care coordination, as well as on the key elements for their sustainability and transferability to other contexts.

The study was carried out in the intervention healthcare network in each country [20]: in Brazil, the Caruaru network (Pernambuco State); in Chile, the Santiago northern network, covering three districts; in Colombia, the Bogotá South-western district network; in Mexico, the Xalapa state network (Veracruz State); Uruguay, the ASSE network of the Western region, covering three districts [27]. The analysis in this study is oriented by the comprehensive conceptual framework on care coordination across care levels described in the Background section.

### Sample

A criterion sample was selected to include all discursive variants on the implementation of the interventions and their results. The following criteria was applied to select informants: stakeholders that had participated in any phase of the PAR process (selection of problems and interventions, design and implementation). Stakeholders could be members of LSC, PP and working groups, other professionals who participated, and managers who provided institutional support. The research team was excluded from the sample. Attendance registers of meetings held throughout the PAR process were used to select informants, who were then contacted and invited to participate. The final sample size ranged from 15 to 41 informants per country (134 in total), depending on when information saturation was reached. A detailed description of the final sample by data collection technique and country is presented in Table 1.

**Table 1:** Final composition of informant sample by country

Type of informant	Brazil			Chile			Colombia			Mexico			Uruguay		
	FGN(n)	II(n)	Total	FGN(***n***)	II(***n***)	Total	FGN(***n***)	II(***n***)	Total	FGN(***n***)	II(***n***)	Total	FGN(***n***)	II(***n***)	Total
Local steering committee		10	10	1 (10)	–	10	–	3	3	1 (4)	–	4		3	3
Inter-level professional platform/working groups	–	–		3 (13)^a,b,c^		13	–	6^a^	6	1 (6)^a,d^		6		3 ^a,c^	3
Middle managers/directors of network	–			1 (3)	5	8	1 (3) ^a,b,c^	3	6	–	3	3		1	1
Health professionals (Level I/II/III)	1 (9)^a^		9	–	8 ^a,b,c^	8	2 (24) ^a,b,c^		24	–	1^a^	1	1 (3) ^a^	8 ^a,b,c^	11
Other professionals/ administrative personnel	–	–			2	2		1	1		–			2	2
**Total**	**1 (9)**	**10**	**19**	**5 (26)**	**15**	**41**	**3 (27)**	**13**	**40**	**2 (10)**	**4**	**14**	**1 (3)**	**17**	**20**

*FG* Focus group, *II* Individual interview, *N* number of FGs, *n* number of participants, *Total* total number of participants

^a^doctors; ^b^nurses; ^c^other health professionals. ^d^Focus group made up of 4 LSC and 2 PP members

### Data collection

Data were collected by means of focus groups segmented by type of informant. Individual semi-structured interviews with the different types of stakeholders were conducted to explore certain topics in greater depth and also when groups could not be formed due to professionals’ shortage of time. Both the focus groups and the individual interviews were conducted using a topic guide, which covered, among other topics, opinions on the contributions of the intervention to care coordination, with particular attention to the mechanisms by which the intervention had its effect, and the necessary conditions and strategies for its sustainability and applicability to other contexts (see Additional file 3 – Interview guide). Only one person refused to participate, in Colombia, giving lack of interest as a reason. The focus groups and individual interviews were held on university premises and at healthcare facilities. Both lasted 45–120 min and were audio-recorded and fully transcribed. Data were collected from November 2017 to May 2018.

### Data analysis and quality control

Thematic analysis [39] was conducted. In the first phase, the results were analyzed separately by country and intervention, using the Atlas-ti or MAXQDA software, and in the second, a cross-country comparative analysis was performed manually. Data was segmented first by country, and then by type of intervention implemented. Recurring themes that appeared in transcriptions and fields notes were identified, coded, re-coded *(data reduction)*. Subsequently, the data were classified through matrices of categories and charts *(data display)*, to detect common patterns by studying regularities, convergences and divergences in the data, explanations and causal flows *(conclusion drawing and verification)*. A cyclical and iterative analytical process of constant comparison was performed, going back and forth between the data and the conceptual framework [39, 40]. The process of category generation was mixed [41] (i.e. the main categories were from the topic guide and the subcategories emerged from the data). The final main categories of analysis were: i) contributions to improving care coordination across care levels (information, clinical management and administrative) and influencing factors related to professionals (interactional factors such as trust, willingness to collaborate, etc.; training; commitment and interest in participating), which were classified in accordance with the analytical framework [20, 42], ii) sustainability of the interventions and conditions and strategies for this and iii) applicability of the interventions to other contexts and conditions for this.

In order to ensure quality of data, triangulation of results was performed by comparing data from different data collection techniques (semi-structured individual interviews and focus groups) and data sources (LSC, PP, professionals and managers). Results were contrasted with previous studies and discussed with informants. The preliminary results were first presented to and discussed with the participants of the interventions – LSC, PP and managers in each country – and later, the cross-country analysis was presented in several international meetings with the participation of all countries. Their views were taken into account in the final analysis. Furthermore, seven analysts (2 international, 5 national) collaborated on the analysis, and differences were discussed until an agreement was reached. These analysts had diverse backgrounds in health and social sciences and in-depth knowledge of qualitative methods, the analytical framework, the research topic and the context to ensure that the contextual characteristics of the local health and social system were taken into account. The researchers gained awareness of their assumptions and preconceptions, as well as their influence on the participatory process, through reviewing the literature, seeking critique from experts and international researchers in the subject, and discussing their assumptions throughout the research process.

## Results

Opinions on the contribution of the implemented PAR interventions to improving care coordination and the factors that influence coordination, as well as observations regarding their sustainability and applicability to other contexts, emerged from the participants’ discourse in each country. The opinions they expressed differed according to intervention. (see Tables 2, 3, 4 and 5 for examples of the analyzed categories and Supplementary Tables S1 to S4 in Additional File 1 for additional quotations). When the informant is a member of the Professional Platform/Local Steering Committee, he/she is identified with the acronym PP/LSC, and otherwise, with his/her professional category.

**Table 2:** Examples of the category ‘Contributions to improving clinical management and administrative coordination’

**2.1. Improved direct communication between PC and SC doctors** - **Encouraged the use of informal communication mechanisms** *“A communication channel was set up on WhatsApp (for endocrinopathies) (...) it was a great help that may have helped to reduce the waiting list (...) Primary care felt more confident about leading* [the care management of the patient]*, because they had the secondary care professional on rearguard”* (Healthcare manager, Brazil) **- Significant change compared to previous lack of communication** *“Up until the moment the intervention started, I’d been working in the institution two and a half years (…) as a specialist and I hadn’t had the opportunity in those two and a half years to share a single academic space or even say hello to a general doctor from other units”* (PP, Colombia) **- Allowed clinical cases on the waiting list or complex cases to be resolved in primary care** *“(..) it was shown to be useful* [joint virtual clinical conferences]*, it’s very useful, for example when the general doctor who has a grandma with dementia that wakes everyone up at 3 in the morning, so he doesn’t just have one patient now but 5, which is the whole family that hasn’t slept… and we* [SC doctors] *can say to him: ‘well let’s have a look at this, or that, we’ll treat it with this medicine, we’ll do these tests, and it’s not necessary for her to come over here’”* (Healthcare professional, Chile) **2.2. Fostered clinical agreement on treatments, diagnostic tests and referral criteria** *“on going over the meeting topics and all that, well let’s just say that we get a space in which to air our differences or opinions, which us general doctors also have on the management of cases (…). So there are discussions, there’s a space for discussion and so somehow more agreements are reached”* (PP, Colombia). *“(…) I think the most positive aspect* [of the videoconferences] *is about coming to an agreement on the type of pathology we’re working on, you know? Through a protocol, so that each one of us understands what they have to do and what their responsibilities are”* (Healthcare professional, Chile) **- Contributed to better control of chronic patients** *“I’ve seen that when patients are managed by the specialist in the same way* [as by the PC doctor]*, their illness improves, their blood pressure readings are more under control, especially diabetic patients. You can see that they’re better controlled because there’s just one sole voice saying what needs to be done”* (PP, Colombia) **2.3. Contributed to reducing unnecessary referrals to secondary care** “*Over the course of this meeting we realised that us general doctors sometimes referred patients that we could manage, and we learnt that from the specialists. You don’t necessarily have to refer hypertensive patients to the specialist. If they prepare us better it might also make their activities more effective, their services may not be so backed up*” (PP, Colombia) **2.4. Improved administrative coordination of patient access across care levels** **- Fostered direct communication to facilitate access to SC and cross-level monitoring** *“We exchanged phone numbers and emails (…) to be able to refer the patient. They generally respond really quickly but there are some cases that are kind of special that always have to be explained, so in those cases we communicate directly with each other”* (Administrative professional, Chile) **- Improved knowledge of administrative pathways and requirements for access to the other care level** *“I thought it was really interesting because it gives us the chance to get to know how the other services work, because we don’t know how they work and we’re suddenly just referring patients over there without knowing (…) and the truth is that the patients are going to waste money on buses and time”* (Administrative professional, Chile) **- Contributed to faster access for patients on waiting lists** *“The latest case was a lady who had an advanced stage of cancer and was in a lot of pain and she hadn’t been referred to palliative care because she’d also been in the private system. So the processes were greatly eased through this and we were able to give her the care she needed”* (Administrative professional, Chile) **- Contributed to a reduction in administratively inadequate referrals** *“(...) various questions would come up* [in the cross-level visits]*: ‘So do you provide this service here? Yes. And the drugs? Look, we’ve got so many people to deal with here, that when you guys send people here to get drugs with a prescription from the hospital, we can’t give them to them, and they keep on coming in’. So they’re also moments of conversation about everyday things, but they don’t get discussed at any other time”* (Administrative professional, Chile) **2.5. Impact limited to the specialities that participated, or little impact on coordination** *“Well yes I think so, in the areas we had contact with. Of course the hospital is huge, there’s a lot of different specialities that also need the chance to get to know each other and that’s the main reservation I have, that one person can only do so much (…) So that’s why I think it may be important to repeat it* [cross-level visits] *to cover more specialities”* (Administrative professional, Chile) *“The relationship between general doctor and the specialist that was directing, leading the progress of the meeting (…) was great because they talked to each other, communicated, they got to ask each other questions and they used tools like WhatsApp text messages. In terms of specialists of the* [whole] *healthcare network with general doctors of the healthcare network, there’s still a long way to go. There’s a huge gap, a persistent void, I’ve yet to hear any news of a first meeting between specialist doctors of the healthcare network and general doctors from primary care”* (LSC, Colombia) *“There have been no changes with regard to referrals at all, nothing’s changed, they don’t make use of the system, two questions* [consultations of specialists by PC doctors] *in so many months...the truth is that it’s precarious”* (PP, Mexico) *“Very few replies* [reply letter in response to PC doctors’ consultations] *were received. Some were replied to so that was good, but if I had to give a general overview of the process, it didn’t work”* (Healthcare professional, Uruguay)

**Table 3:** Examples of the category ‘Contribution to improving factors that influence coordination across care levels’

**3.1. Improvement in interactional factors between health professionals in the network** **- Helped to get to know professionals of the other level and their work context and fostered personal connections** “*I’ve got the impression, as I also participate in the consultations and videoconferences, that ties have been made between primary care and the specialists (…). We often have that frank conversation of: look, this is what’s going on, or this is the real case scenario, as seen from primary care and also as seen from the hospital perspective (…) so that leads to this kind of ‘Ohhh, right! Well let’s see how we can put our heads together and solve this then’”* (Healthcare Manager, Chile). *“(…) despite having worked a long time in the network, they didn’t know their counterparts, at the hospital level in this case (…) We noted a high level of interest among the staff in participating in the implementation of this activity* [cross-level visits]*”* (Healthcare manager, Chile) “*From the specialist point of view, we started to understand a bit more about the limitations of patient management and diagnosis at the primary level. It wasn’t necessarily that the general doctors didn’t know how to manage the patient”* (PP, Colombia) **- Improved opinion of the other care level** *“Interpersonal relationships, and interprofessional ones too. Creating empathy, putting yourself in the other person’s shoes (...) understanding the problems of many professionals in health care, and how we were going to come together to achieve it”* (Healthcare manager, Brazil) *“When we started these meetings, seeing as we wanted to improve communication between levels, we noticed that general doctors had a lot of preconceptions about specialist doctors, and specialists about general doctors, which we started to discuss and we realised that a lot of those preconceptions were wrong”* (PP, Colombia) **- Improved trust, willingness to collaborate, communication between participants** *“Yes, the specialists are always on hand, they’ll give you their email, not their personal one but the work one, of the unit, and they are available to chat about complicated cases and all that. So that’s been really good”* (Healthcare professional, Chile) *“After that they felt more at ease in making queries, and that the doctor could call the specialist, the internal medicine doctor in this case, and consult them on something”* (LSC, Colombia) *“It’s important that regular contact is maintained with the specialist doctors because that builds trust, integrates the care levels, ties us closer together and makes it easier to manage the patient’s care pathway, I mean without feeling so apprehensive and knowing they can refer them back again [if necessary]. That mustn’t be lost”* (PP, Mexico) **- The PAR process improved interactional factors between managers of the network** *“(…) Hospital: “There’s nothing more I can do because primary care is doing such a terrible job”. Primary care centre: “There’s nothing more I can do because the hospital is doing such a bad job” (…) And with that we don’t get anywhere constructive (…) I would say that this in some way....means we basically see ourselves as one and the same. So we’ve really managed to lower our defensive barriers (…) and start to understand that what we really need to do is help each other out”* (LSC, Chile). *“it’s like a sign that a shared vision of the network is appearing, which was the problem we had in the first place (…) Of course, and people perceive it and everyone can feel it now. If I’ve got a problem I know who to speak to and I know they’re going to help, you know? And the certainty that they’ll help me, that they’ll collaborate with me for the sake of the patient”* (LSC, Chile) **3.2. Improved training/updating of PC and SC doctors** *“We acquired a greater ability to resolve pathologies. You generally come in fresh out of medical school and then you don’t keep up a formal study routine, or get like an update on things, so you stick with how things were when you graduated, and carry on working in the same way for the next 10, 15, 20 years”* (PP, Colombia) **- The PC doctors who participated became sources of reference in their units** *“(…) they* [participating PC doctors] *turned into sources of reference on patient management in their health centres (…) the other general doctor could ask them when they had a query or concern about clinical management”* (LSC, Colombia) **- Contributed to improving PC doctors’ management of patients and the appropriateness of referrals to SC** *“We’re managing to gradually reduce the number of hospital referrals, (...) anything to do with surgical specialities has to be sent over, there’s no way round that, but we’ve been able to solve a lot of things though this, you know?”* (Healthcare manager, Chile) “*When they give this type of training, it’s great, because they focus precisely on common problems that come up all the time, that we ourselves see and that we’re helping to transmit to the hospital. ‘Oh, yeah right! I had a patient like that, I didn’t manage it right’, and so now we go back to primary care level and improve on that way of treating the patient”* (PP, Mexico) “*As a result of this* [training] *it turns out they* [the patients] *have come in with their case management well established and they pretty much go straight to the hospital without wasting any time. In obstetrics, every minute and second counts*” (PP, Mexico). **3.3. The PAR process fostered professionals’ interest in participating in the interventions** *“I think that makes it more credible, because it’s not a structure, it’s something you gradually shape (…) to all the needs that arise in the moment. Some get involved (…) and that makes people start to feel part of it, they feel motivated (…) we’re building up motivation throughout the whole process. That’s been the most wonderful thing that this whole process of implementation and design has brought”* (LSC, Chile)	

### Contribution to improving care coordination

Opinions on the contribution of interventions to improving clinical coordination and factors that influence coordination varied according to the intervention type and country. Cross-level joint meetings were considered to have made an important contribution to care coordination in Chile, Colombia and Mexico, although some informants also pointed out that the impact was limited to the coordination between PC and the medical specialties that participated in the intervention (Table 2 and Supplementary data). Similarly, in Chile informants highlighted the contribution of cross-level bidirectional visits between PC and SC to improving the administrative coordination of access between care levels. In contrast, no noteworthy contribution to coordination was perceived for offline virtual consultations in Brazil or Mexico, nor the referral and reply letter in Uruguay, due to professionals’ low level of use of these interventions (Table 2.5 and Supplementary data). In general the improvement of coordination-related factors was attributed to the PAR process, with varying intensity in the informants’ discourse according to country.

#### Contribution to improving coordination of patients' clinical management

Several contributions of the joint meetings to clinical management coordination emerged: improved direct communication between PC and SC doctors, with particular intensity in the discourse of professionals in Chile and Colombia, and somewhat less in Mexico and Brazil; improved clinical agreement between PC and SC, especially in Colombia; and more appropriate referrals.

The joint meetings improved *direct communication* between participating PC and SC doctors for consultation and feedback on queries regarding patient follow-up and referral, not only during the meeting itself, but also through an increased use of less formal means of communication such as WhatsApp or phone (Table 2.1). Some informants in Colombia and Mexico found that there had been a considerable change to the previous lack of cross-level communication.

Most informants in Chile pointed out that as a consequence of the virtual joint meetings, agreement was reached on how to manage or resolve clinical cases that were complex or on the waiting list, thus avoiding referrals to specialist care. In Colombia and Mexico, they also highlighted solving queries and reaching general clinical agreements on disease management, not only for specific cases: *“(...) so the fact that, for example, I can now talk to the dermatologist and say “Look, I’ve got this case...” Before the videoconference that just didn’t exist and was unthinkable. So, now I’ve resolved a couple of cases that were really complicated, and there was a waiting list of I don’t know how many years (…) so in that sense it’s been really favourable (…)*” (PC doctor, Chile).

Likewise, and in direct relation to the above, informants highlighted the establishment of *clinical agreements* (on treatments, diagnostic tests and referral criteria) stemming from the joint discussion of clinical cases and training sessions as one of the most significant contributions of joint meetings, particularly in Colombia (Table 2.2). Here, and also in Chile, they pointed out that the joint meetings allowed them to adapt or establish joint care protocols taking the local context into account (e.g. restrictions on resources), *“(…) One thing is the guidelines, another is what you see in the conferences...they haven’t been adapted to the context of our population, which is a vulnerable population, to our resources. What have we got? What paraclinical tests, what medications have we got? So that’s where the strength of this strategy lies*” (PP, Colombia). A more adequate control of chronic patients emerged as the main consequence of unifying the criteria of cross-level care.

Lastly, improvements emerged in the *appropriateness of referrals* to specialist care as a result of joint meetings in Chile, Colombia and Mexico, and of shared care guidelines in Brazil (Table 2.3). The informants noted a reduction in unnecessary referrals as a consequence of having strengthened the capacity of primary care to resolve cases (through giving PC doctors better training), and reported more agreement on the referral criteria through these interventions. In Mexico and Brazil they also highlighted the early detection and timely referral of patients with complications: “*I’ve already seen that, because of the patient referral centre, the number of people heading to nephrology has increased considerably. Because they [PC doctors] have started to give diabetic patients nephrological care. That’s fantastic!* (Healthcare professional, Brazil).

#### Contribution to improving administrative coordination

According to the majority of informants in Chile, the cross-level bidirectional visits between PC and SC improved administrative coordination between levels because they permited direct communication between professionals to facilitate access to specialist care and monitor the patient’s transition between levels, joint problem solving in administrative matters, and a better knowledge of the administrative pathways and access requirements of the other care level (Table 2.4): *“(…) we started to get to know the secondary level problems over there and they got to know the problems we face here. We started to realise that we had nearly the same problems and that there were lots of things we could solve. That also facilitated almost direct communication, one on one, the person in charge of one thing here liaising with the person in charge of the same thing there (...) so it flowed better, shall we say, the care of the patient”* (Healthcare Manager, Chile). Informants reported that the improved administrative coordination helped to reduce waiting times and administratively deficient referrals (incomplete information, etc.) and to provide the patient with more adequate information on the procedure.

#### Contribution to improving the factors that influence coordination

Both the joint meetings and the PAR process through which they were designed and implemented, contributed to improving the interactional factors of professionals of the different care levels, and the former also contributed to their training (Table 3.1). The first point to emerge from the informants’ discourse was that they got to know both the professionals and the working environment (available services, functioning, and difficulties) of the other level better, and personal connections were made between professionals of different levels, which was stressed with particular intensity in Chile. Knowing each other in person, according to informants, helped to improve their opinions and understanding of the other care level, increased their willingness to collaborate and find joint care strategies, facilitated direct communication for patient follow-up, and in Chile, moreover, bolstered their feeling of belonging to the network: *“… barriers are now being broken down between the specialist* [and the general doctor]*, ‘because I’m the one who knows the most and the general doctor has no idea’. So now the specialist is starting to see the reality of a general doctor’s work with all its limitations (…) That’s when they open up their world view and say, ah right!, well my suggestion would be this, or whatever other path that allows the patient to improve their condition and their quality of life”* (Healthcare Manager, Colombia); “*I think there’s certainly been a great contribution to coordination. For a start, this situation of acknowledgment…, this situation of acknowledgement of the different parts (...) And I think that this respect that has kind of been generated between one and the other paves the way, it creates the chance to build something together*” (Healthcare Manager, Chile). In Chile, this improvement in interactional factors – personal relationships, trust – also occurred among administrators and middle managers that participated in creating the cross-level instruments to implement the interventions (LSC, PP, cross-level working groups), and spread to other instances of cross-level management already present in the network.

According to informants, especially in Colombia, joint meetings contributed to better training for PC doctors – turning them into useful sources of reference on particular topics in their units – and also for the specialists who acted as facilitators (Table 3.2). This helped to improve PC doctors’ management of chronic patients (greater ability to diagnose and prescribe the right treatment) and the appropriateness of their referrals to specialist care: *“Thanks to that update and the contextualization of the diseases, therapeutic skills were improved, and resources were optimised, because on that basis we can see what paraclinical tests we can request, according to the level of intervention, what should we be doing for patients with hypothyroidism, with diabetics...should we request glycated hemoglobin? Yes or no, and how frequently”* (PP, Colombia).

According to informants in nearly all the study countries, the participation of professionals in the PAR process (problematization, selection, and design and adjustment of interventions) also modified other factors that influence coordination (Table 3.3): the growing awareness of the significance of the problem of clinical coordination in the network and the need to intervene, and – particularly with regard to joint meetings in Chile, Colombia and Mexico – the selection of interventions that are appropriate to perceived needs. These factors fostered greater commitment and interest in participating in the interventions.

### Sustainability of the interventions and conditions and strategies for this

The majority of participants from all countries voiced the opinion that the implementation of the intervention should continue (Table 4.1). Joint meetings, and even more so the virtual consultations in Chile, were considered sustainable because they were institutionalized; in other words, they were built into the network planning and programming guidelines of the Health Department. In Chile, Colombia, and México to a lesser degree, several elements were identified that favoured the sustainability of the joint meetings: the intervention being in alignment with or incorporated into local and/or national network policy, the results obtained in improving coordination, and the interest of professionals in the interventions (boosted by their participatory nature). *“It’s a strategy that allows us to strengthen our professionals within the same integrated healthcare model, it favours the model in a very positive way, working together as a team. The model has become a real ally with this strategy”* (Healthcare Manager, Colombia); *“(…) For the hospital it’ll be really important to establish it and keep it going, won’t it? (...) From a healthcare perspective there are only gains to be made, because this patient here saves himself the 1500 COP (0.41 USD) on the bus to come all the way here, and stand in line for who knows how long. If his doctor can hand him the solution right there in his health centre that’s fantastic, I can’t see any downside (…) you’d have to be barking mad, as they say around here, to be against such a great initiative”* (Healthcare professional, Chile). However, threats and difficulties were also perceived for the sustainability of the interventions, mainly with regard to joint meetings in Colombia and cross-level visits in Chile (Table 4.2). The obstacle that emerged in Colombia was the limited understanding of top management of the potential benefits of the intervention in terms of the overall costs of healthcare in the network, and in Chile, the limited involvement of the network coordinator (Health Service) in its organization and institutionalization as a result of an imminent change of government. The change of government was also seen as a threat; in Mexico in particular due to the tradition in politics of new governments scrapping the projects of the outgoing administration: “*One of our governmental authorities’ bad habits, at all levels (...) ‘get rid of it and let’s start over from scratch again’. And it’s the same every 6 years or every 3 (...). So - everything I’ve done was for nothing? (...) you’re not going to keep it going?”* (LSC, Mexico).

**Table 4:** Examples of the categories of ‘Sustainability of the interventions and conditions and strategies for this’

**4.1. Sustainability of the interventions** **- Desire for the implementation of the interventions to continue** “*What we hope is that the strategy will continue, that there’s no obstacle big enough to make it cease to exist, and that hopefully it can be rolled out in other places. The benefits are so great, when I talk to doctors from other networks that don’t have this space* [joint meetings] *...and it’s difficult to attain, and now that we’ve attained it, well we mustn’t lose it”* (PP, Colombia) **- Institutionalization of virtual consultations** “*I think that including these consultations in the planning is something that has undoubtedly favoured the rollout of these events. That we now have instructions and previously allotted times to dedicate to this kind of activity, that was a step that really facilitated participation”* (Healthcare professional, Chile) - **Facilitators of the sustainability of the joint meetings: alignment with/incorporation into state policy, favourable results and professionals’ interest in the interventions** *“Because they’re also very much in keeping with what the ministry authorities, more than any one particular government, have been promoting. It’s in line with telemedicine, with the optimization of the network”* (Healthcare manager, Chile). *“The intervention itself should be maintained at the healthcare network level because when you read over what’s set out in the integrated health model, in part 9, in the model on human talent development, it states the need and even outlines the set of problems encountered regarding the level or quality of training that health personnel currently get (…) So this strategy becomes an opportunity”* (LSC, Colombia) “*Yes, totally sustainable, I mean, the main point is the productivity of doctors, but we have to look beyond productivity… the outcomes*, *not in the short term but in the medium to long term that this can have in terms of avoiding complications for the patients. It’s not unknown that having trained and happy staff will facilitate any job, so, we’ve got to look a bit beyond the money …*” (PP, Colombia) **4.2. Threats or difficulties for the sustainability of the joint meetings** **- Lack of management’s support for their maintenance** *“I think that the managers don’t see it as being something productive for them... the management doesn’t see that the training of professionals is important as a way of improving the financial side, so they have to see clearly that unifying criteria reduces costs, when they see that as important, they’ll promote those spaces, but if they don’t think that has an impact, it’s going to be difficult”* (PP, Colombia) **- Lack of institutionalization of cross-level visits** *“For them to be continued, they’re not yet (...) an institutionalized tool, so for that they need to be included as part of the induction strategies that should be promoted by the services and by the establishment’s training or recruitment department”* (Healthcare manager, Chile) **4.3. Conditions for the sustainability of the interventions** **- Institutionalization of the interventions** *“(…) it depends a lot on the government that’s currently in power, it depends on public policies, it also depends on* [political] *will,* etc.*, however this work should be established as a type of protocol, that every so often we could roll out this training or deliver this information, especially for new people”* (Healthcare professional, Chile) **- Institutional support** *“I think it’s essential that the managers continue to incentivize this, give it their support, you know? Because that communication is important”* (Healthcare professional, Brazil) **4.4. Strategies for the sustainability of the interventions** **- Incorporation of the interventions into network planning/local policy** *“basically it has to be a policy of the management, they have to see the importance, the need for these spaces for training and communication between specialists and general doctors. If not, then it’s really difficult, we go back to how we were before”* (PP, Colombia) **- Allocation of resources** *“(…) they have to define the structures, the processes and of course the resources to make it sustainable (...) to a large extent all they really need to do to is find a way to bring it all together in order to institutionalize it”* (LSC, Mexico) **- Appointing staff to take charge and defining their roles in the implementation process** *“so the idea is to maintain those connections, right? And that this becomes like a working method* [PAR]*, that it’s not only...like at the moment, that it relies on people’s good will, because maybe tomorrow, I don’t know, XXX leaves (…), so then we have to put someone else in. I retire, another person comes in (...) I mean the idea is that this is what we do, so it’s part of the role, it’s part of your function, it’s part of your job to do this, this is just one more of your duties, it’s not like it’s just out of sheer good will”* (Healthcare manager, Chile) **- Making processes and spaces official** “*I think it’s important to make progress in systematizing and making the processes more official, (...) so that if someone were to say, ‘no, maybe we should scrap it* [the intervention]*’(...) that things are more...official* [in terms of processes, roles, resources, etc] *so that it blocks that in some way”* (LSC, Chile) **- Raising awareness of the usefulness of the intervention through disseminating its results and evaluating its cost-effectiveness** *“start to get them* [healthcare managers] *involved, start to tell them, ‘look how important it is’. And then show them the results (...) that’ll motivate them a bit, and also motivate them in terms of the benefits it’ll bring for the patients and for themselves”* (Healthcare manager, Colombia) *“Because they haven’t translated it into numbers, they haven’t put it into financial figures… because it’s expressed in figures like adherence to patient care protocol, coverage, commitment to care … but if they published financial data, they would see the importance”* (PP, Colombia)	

In line with the above, the additional elements identified by informants in order to sustain the interventions, and the PAR process in Chile, were institutionalization and institutional commitment (which must be renewed with every new management) (Table 4.3). In terms of institutionalization strategies, they cited the incorporation of the intervention into network planning, the allocation of resources (mainly professionals’ time to be able to participate), delegating the implementation of the intervention to specific network staff members and defining their roles, and officially designating procedures and spaces (Table 4.4). *“(…) Until now we’ve been sort of doing it out of good will (…), and it’s an experiment. I think it should be made official that, for example, it should be counted as a medical activity within a doctor’s normal working hours, same for a nurse (…)”* (Healthcare professional, Chile). To garner the support of managers, particularly in Colombia, it was suggested to spread awareness of the usefulness of the interventions by disseminating their results and cost-effectiveness.

### Applicability of the interventions to other contexts and conditions for this

Given the results obtained through the joint meetings in addressing common coordination problems in healthcare networks, and their alignment with/incorporation into local or national network policy, the joint meetings were deemed applicable, or their use was recommended, in other contexts (Table 5.1). In Mexico, they had already been replicated in other state-owned networks. Informants believed the PAR method to be especially suitable because it allowed them to identify interventions that met the needs of each particular network: *“I don’t know if the other municipalities have the same problems. It was our choice, communication between professionals. However, as soon as you start to discuss, reflect, [...] you move out of your comfort zone a bit. I think the other municipalities could benefit greatly”* (Healthcare professional, Brazil).

**Table 5:** Examples of the categories ‘Applicability of the interventions to other contexts and conditions for this’

**5.1. Applicability of the interventions to other contexts** **- Interventions are applicable, their application is recommended** *“I find it such a delightful tool to use that it comes easily…getting other people enthusiastic about using it (…) you avoid all the* [patient’s unnecessary] *trips* [to SC]*, you can even create meetings, not with the whole network, but with one or two practices, so the flexibility it offers (...), it sells itself (…) people start to join in quickly...”* (Healthcare manager, Chile) **- They contribute to tackling a common problem in different settings** *“Yes, of course, it’s very viable. Because we need this kind of strategy, for the impact it has and because that communication between the specialist doctor and the general doctor is necessary. That’s a common problem in all the networks and we all need to improve on patient care”* (LSC, Colombia) **- They are aligned with/incorporated into local or national network policy** *“(…) the fact that the project has reached Health Department level, and that they’ve incorporated these interventions and their basic structure* [into the programming guidelines of the Health Department]*, means that others* [other healthcare networks] *can adopt them and put them into practice”* (LSC, Chile) **- Benefits of replicating the PAR method** *“Obviously they can be replicated in other spaces but the model to follow is participation.”* (LSC, Chile) **5.2. Conditions for the applicability of the interventions to other contexts** **- Interest of professionals and managers in improving care integration** *“I think there is interest in other regions, other professionals wanting, wishing and not knowing how to take that approach* [PAR]*. But the manager has to facilitate it, the health departments [...]”* (Healthcare professional, Brazil) *“(…) a bit of political will, it should be taken to other places and there should be the will to implement it, and [they should] always be stressing the importance of this, of coordination”* (PP, Uruguay) **- Institutional support in the allocation of resources needed for implementation** *“(…) giving the healthcare teams in primary care centres time to be able to attend and the technical capacity: computer, microphone, whatever they need. I think that the professionals would have no problem with acquiring more knowledge, especially from a specialist, especially if they’re going to help you coordinate things”* (Healthcare professional, Chile) *“If they do another project at some stage, they should take into account the real resources they have. Improving resources of all kinds, I think that would help the projects to be more genuine”* (PP, Uruguay) **- Respecting the basic elements of PAR when applying the method** *“(...) discussing with people, with the managers, with the professionals. Follow the same process that you did here”* (Healthcare professional, Brazil) **- Improve the design and implementation of offline virtual consultations and referral forms** *“If you make them* [offline virtual consultations] *simpler, more direct, with clearer communication between the people you want to do them, the results will be seen more quickly and the system of consultation or interaction should work”* (LSC, Mexico) *“It should be continued into the medium term, it should be included in the electronic medical records and it shouldn’t be on paper”* (LSC, Uruguay)	

Informants widely agreed that the motivation of professionals and managers to improve coordination across care levels was the key element to applying the interventions in other contexts, together with institutional support in the allocation of resources, a stable network leadership committed to the implementation process, and adherence to the basic components of the PAR method in its implementation (Table 5.2): *“I would add that replicability is feasible (...) the most important thing is absolute perseverance and our* [the LSC’s] *conviction, I mean the conviction of those at the helm, that it really is them at the front leading* [the process]*, that they really are going to be the ones getting the rest on board”* (LSC, Chile).

Lastly, with regard to interventions with less influence on coordination – offline virtual consultations, use of referral and reply letter – the participants also expressed a wish for them to continue, and deemed their application in other networks viable, but pointed out that changes would be required in their design and implementation to encourage its adoption by professionals (Table 5.2).

## Discussion

There is a growing consensus on the need to explore alternative ways of implementing integrated care strategies that can lead to better outcomes [1, 30, 43]. This qualitative study contributes to improving our understanding on the use of a participatory approach to introducing interventions to improve coordination across care levels, an approach not commonly used in this area [30] despite its reputation as an effective means for achieving organizational change and closing the gap between theory and professional practice [5]. Although this study does not aim to generalize the results from a statistically representative sample, important lessons can be drawn for the applicability of similar interventions in other contexts, from the process of generating ideas that come from in-depth analysis of concrete experiences [44] in five different contexts in Latin America.

The results show that interventions such as joint meetings based on reflexive methods, which are selected, designed and implemented locally through a participatory process, contribute substantially to improving the clinical management coordination elements relevant to each context, as well as the determinants of interrelation between professionals. These findings are consistent with a great amount of evidence from implementation science [9–11, 13] and, to a lesser degree, on care coordination mechanisms [30, 45–47] that highlights the importance of adapting the design and implementation of interventions to the local context (taking into account problems, priorities and contextual factors) in close collaboration with the personnel involved, in order for them to be effective.

The cases in which informants could barely identify any contributions to the improvement of care coordination – offline virtual consultations and use of the referral and reply letter – appear to be related to both contextual barriers and deficiencies in the PAR process and in the content of the interventions [33], aggravated in some cases by a short implementation period, which resulted in low uptake of these mechanisms. An appropriate application of the PAR process in a favourable context also emerged across the board as a necessary condition for the extension, sustainability and replicability of the interventions in other contexts.

### Joint meetings between levels: wide-ranging results on the clinical management and administrative coordination, varying according to the needs of the context

It is striking that such an apparently simple intervention – meetings between professionals, in person or online, to find solutions together to the problems of coordinating patient care, whether the purpose is to discuss clinical cases, give training, establish joint protocols or the induction of staff into the network – should obtain such wide-ranging and diverse results in the improvement of clinical management coordination (communication in follow-up, clinical agreement, appropriateness of referrals) and administrative coordination. This may be related to the PAR approach adopted in the design and implementation of these interventions and the reflexive and participatory methods used during the sessions, which entailed joint reflection on cross-level working practice aimed at reaching agreements and the continuous evaluation and adjustment of methodology and content [33]. Firstly, the professionals’ participation in the problematization of the fragmentation of the network facilitated their engagement, as they were tackling a problem they felt to be their own. Secondly, in keeping with PAR studies in other areas [14], it strengthened the professionals’ interest and motivation to participate [33]. Informants’ observations that the impact on care coordination was limited to the specialities involved point to the need to ensure that the intervention reaches further (to include other medical specialities in which cross-level clinical coordination is necessary to improve quality).

One of the most significant findings of this study was that the PAR process allowed the content of the interventions, and thus the results obtained, to be progressively adapted to the needs perceived by participants, enriching it with elements that had not been contemplated initially, such as the improvement of administrative coordination of access between levels in Chile. Although similar interventions – joint meetings – were chosen across the different contexts in order to tackle common problems (lack of communication and clinical agreement between levels), as they were being implemented, more specific difficulties of their daily practice were also tackled along the way, meaning that the contributions to coordination varied according to the setting. Thus in Colombia, for instance, more emphasis was placed on medical training and clinical agreement in the management of patients in order to address restrictions at the primary care level (diagnostic tests and drugs that can be prescribed), in the context of a health system based on a market model that incentivizes insurers and public providers to minimize costs [48]. In Chile, it was resolving cases on SC waiting lists, with long waits, in the context of a health system with severe problems of access to SC, especially for non-priority illnesses (those excluded from the Explicit Guarantees in Healthcare (GES) plan) [49]. In Mexico, the focus was on the timely referral of high-risk pregnancies and adequate follow-up at primary healthcare centres, in the context of a high maternal mortality rate in the state of Veracruz [50]. The focus in Brazil was on extending the intervention to mental health, in the context of an increase in prevalence of illnesses of this type. Other studies also highlight the flexible, dynamic and locally adaptive nature of PAR interventions [14, 51], which lends greater relevance and validity to the outcomes achieved when responding to problems that professionals face in their daily working practice.

In the specific case of joint clinical case conferences, most studies identify their main contribution to be a reduction in the number of face-to-face patient consultations and physical examinations carried out in secondary care [52–54]. Informants in this study highlighted another contribution more forcefully: improved direct communication, which helps not only to reach agreements on the follow-up of patients, in cases of high complexity or uncertainty [24, 55], but also to adapt protocols to local conditions, in the sense that it fosters knowledge of the working environment of professionals of the other care level and joint efforts to find strategies for patient follow-up. In Chile, the results show that direct feedback mechanisms also facilitate the coordination of access for patients on SC waiting lists, against a backdrop of significant structural and organizational barriers to access.

Nevertheless, although feedback mechanisms such as joint meetings are more suitable for addressing selected coordination problems, by generating direct communication spaces in which to solve common or complex problems, in order to also guarantee equity of access, it is essential to roll out the interventions across the whole country, and to make them more formal or official (priority protocols, standardization of communication channels, etc.).

### Joint meetings that foster personal relationships, mutual trust and training of professionals

Direct (face to face) contact between professionals of the two care levels, together with the reflexive method used in the sessions [33], helped to improve factors related to professionals which, according to previous studies [27–29, 42, 56], influence coordination between care levels. Firstly, mutual trust improved, and favoured the creation of personal connections and horizontal relationships, bypassing the established hierarchies of levels and professions. This improved interaction between professionals, also reported in other PAR studies [57], may also have had an impact on the use of other existing coordination mechanisms in the network. These results therefore lead us to reflect on the advantages of promoting physical meeting spaces for co-workers in a context of increasingly asynchronous means of communication – electronic medical records, offline virtual consultations, e-mail – in which opportunities for direct contact are severely reduced [58].

Secondly, the professionals that participated in the joint meetings in all the different experiences reported an improvement in expertise and capacity to resolve cases, particularly but not exclusively among PC doctors, a finding in keeping with several systematic reviews of both PAR studies in health services [5, 14, 17, 59] and ongoing training programs [46, 56, 60, 61] of similar characteristics. Reflection on working practice in a PAR approach is considered especially suitable as a tool for training and professional development in complex dynamic situations, to reduce the gap between theory and practice, and to attain a sustainable educational effect on users [14]. This contribution emerges with particular intensity in Colombia, probably due to the exceptional nature of this type of training-oriented intervention in the network studied as a result of incentives to minimize costs [62], and to the fact that in this experience the reflexive method was applied in greater depth [33].

### Importance of implementing the PAR process in a favourable context for the sustainability and applicability of the interventions to other contexts

The PAR approach is considered to be an effective way to implement sustainable changes in organizations, although more studies of a longer duration on this aspect are required, as well as more sources of funding to be able to carry them out [14, 59, 63]. From the discourse of the informants a greater certainty emerged regarding the sustainability over time of the joint meetings, especially virtual consultations in Chile, which at the time of analysis had already been institutionalized and extended to more centres and specialities. This is probably related to the fact that in this experience, the PAR design and implementation process was carried out in a longer, more in-depth and participatory way, fuelled by favourable contextual factors [33]. It is precisely the convergence of these contextual factors and the participatory process that emerges in the discourse of the participants in the different experiences, highlighted as necessary elements for the longevity and sustainability of the interventions in the network and replicability in other contexts: the interest of professionals and managers, institutional support, visible results and an appropriate application (time, method and levels of participation) of the PAR process [33]. This study therefore shows that merely taking a participatory approach is not enough to guarantee the sustainability of the intervention; it also requires institutional support and commitment, and needs to be institutionalized in the network (incorporating it into planning, allocating resources and appointing a person to take charge), especially in contexts of high institutional instability commonly found in low and middle-income countries [64]. The challenge lies in institutionalizing the intervention, while at the same time preserving the essence of the flexible, reflexive and democratic PAR method and giving continuity to the spaces for reflection created in the process.

## Limitations of the study

In Brazil, Mexico and Colombia, the turnover of members of the LSC, PP and managers prevented informants from forming a complete and, in some cases, recent opinion on the perceived results of the interventions, as well as key aspects for their sustainability and transferability to other contexts. Although this limitation was tackled by carrying out interviews with participants in each phase, it may have had repercussions on the depth of the data collected. The short duration of the implementation stage within the time frame of the project in the case of some interventions contributed to informants’ perceptions that the effects on coordination were minimal, thus, further evaluations are required to analyse the long-term impact.

## Conclusions

The results of this study show that interventions designed and implemented at a local level through the PAR process, such as joint meetings based on reflexive methods, contribute substantially to improving coordination across care levels and strengthening the capacity of primary care to resolve health problems in the follow-up of patients. These results are attributed both to the participatory process and to the type of intervention based on mutual feedback, which firstly enabled networks to adopt interventions adapted to the local needs as perceived by its professionals; secondly, promoted joint meetings between professionals of different care levels to discuss their practice, generating greater interest and commitment to the interventions; and lastly, improved mutual trust between care levels, fostering a willingness to collaborate. The scarce contribution to care coordination, in contrast, of the other interventions, also highlights the influence of the context on the PAR process, the relevance of the quality of the participatory process, and the adequacy of the content. The PAR approach, therefore, when applied within a suitable time frame and with sufficient resources, can serve as a valuable component in strategies to improve coordination between care levels, both in the region studied and in other contexts.

## Supplementary information

**Additional file 1.** Additional examples of the analyzed categories. Description of data: additional examples (quotations) of the analyzed categories in the original language (Spanish and Portuguese).

**Additional file 2.** Table 1. Characteristics of the implemented interventions in the study networks. Description of data: Description of the characteristics of the implemented interventions.

**Additional file 3.** Interview guide. Description of data: Topic guide for the analysis of contributions of the interventions to care coordination, necessary conditions and strategies for its sustainability and applicability to other contexts.

## Data Availability

The dataset (which includes individual transcripts) is not publicly available due to confidentiality policies. However, more examples of the analyzed categories are included as supplementary material (Additional File 1).

## Abbreviations

PAR: Participatory action research
LSC: Local steering committee
PP: Inter-level professionals platform
PC: Primary care
SC: Secondary care
