# Supplementary material for: Can care coordination across levels be improved through the implementation of participatory action research interventions? Outcomes and conditions for sustaining changes in five Latin American countries
Source: BMC Health Serv Res. 2020 Oct 12;20:941. doi: 10.1186/s12913-020-05781-7 (PMC7552474; doi:10.1186/s12913-020-05781-7)
Supplement: Supplementary file 1 — Additional file 1. Additional examples of the analyzed categories. Description of data: additional examples (quotations) of the analyzed categories in the original language (Spanish and Portuguese). [file 12913_2020_5781_MOESM1_ESM.docx]

**Additional examples of the analyzed categories**

| **Table S1: Examples of the category ‘Contributions to improving clinical management and administrative coordination’** |
| --- |
| **1.1. Improved direct communication between PC and SC doctors**  *“(...) lo principal es la comunicación, que nos facilita la comunicación con los especialistas para poder definir pacientes y disminuir la lista de espera. También porque hay muchos pacientes que están en lista de espera de hace mucho tiempo no se ha podido definir los manejos en espera de una valoración por una especialidad (...). Además de capacitarnos en temas específicos, también nos ha servido para eso aclarar dudas y poder solucionar de pronto pacientes que lo podríamos hacer como una comunicación directa, como una especie de tele-medicina”* (Healthcare professional, Chile)  - **Encouraged the use of informal communication mechanisms**  *“(…) esa videoconferencia que se hizo, fue la primera como acercamiento que tuve más a APS. Mañana tengo otra videoconferencia, así que ya la estamos preparando. (…). Entonces igual tenemos ya una mayor relación, conozco más los nombres, y también se han hecho Whatsapp de cada comuna. Entonces de repente cuando vienen consultas o casos clínicos, me mandan un mail o me escriben por Whatsapp* (Healthcare professional, Chile)  *“Hay que mencionar que los asistentes a la plataforma, siempre tuvimos un canal de comunicación muy bueno dentro de la plataforma y fuera de ella. Fuera de ella a través de todas las ayudas de comunicación tecnológicas; en muchas ocasiones me escribían al Whatsapp, que un caso clínico. Incluso les ayudé con el tema de referencia, cuando tenían dudas de un paciente que requería hospitalización”* (PP, Colombia).  “[A raíz de la capacitación conjunta] *a mí me llaman directamente al celular, me mandan un mensaje y entonces… ‘”ye soy fulanito de tal, de tal centro de salud… doctor, tengo una mujer, una paciente así y así, ¿se la mando?. ¿Qué hago, que le puedo hacer?”. Hay ese contacto muy estrecho*” (LSC, Mexico)  **- Significant change compared to previous lack of communication**  *“O sea, yo encuentro que al hacer la videoconferencia es mucho más disponible para todos, porque no es factible para todos reunirse en un lugar. Entonces quizás se llega a más personas.”* (Healthcare professional, Chile)  *“Aquí capacitaciones son esporádicas, la actualización del saber médico no es continua, es demasiado fraccionado y eso también influye en la calidad de la atención. A uno le exigen calidad, pero resulta que no lo están capacitando, no lo están actualizando sobre los temas. Entonces le toca a uno por su propia cuenta hacerlo. Entonces, esa es otra ganancia más”* (PP, Colombia)  *“*[El contacto que se generó con los médicos] *fue a raíz* [de las reuniones conjuntas], *porque ni siquiera me conocían, ni siquiera tenían mi número de teléfono directo”* (PP, Mexico)  **- Allowed clinical cases on the waiting list or complex cases to be resolved in primary care**  *“Acho que resolutividade. Quando a gente traz [...] o número de casos que a gente tem, e quando as duas (AP e AE) conseguem conversar, consegue resolver”* (Healthcare professional, Brazil)  **- Improved communication during medical training**  *“Sí, siento que sí funcionó* [la capacitación conjunta]*, porque para mí, el tener la comunicación directa con los especialistas en las reuniones, sí fue eso de conocernos y poder resolver en algún momento algunas dudas o tratar el mismo tema de la paciente que trabajamos en común. Pues sí nos ayudó, como que hubo esa comunicación, desde ahí de manera directa y hasta cierto punto pues con las capacitaciones también nos ayudaron bastante”* (PP, Mexico)  **1.2. Fostered clinical agreement on treatments, diagnostic tests and referral criteria**  *“La mayoría de las reuniones llegamos a conclusiones mutuas, a pesar de discusiones un poco largas casi siempre llegamos a acordar cómo íbamos a enfrentar un problema clínico: qué exámenes hacer, cuál no íbamos a hacer, cuáles considerábamos después de las reuniones que ya no eran relevantes o cuáles sí tenían que hacer los médicos generales que quizás tenían bloqueados por el sistema de salud”* (PP, Colombia)  **- Facilitated the adaptation of joint protocols to the local context**  *“*(…) *el caso más emblemático, a mi parecer, es el de Nefrolitiasis, que estableció un protocolo práctico, muy sencillo, y que al final uno puede aplicar en el box todos los días. Cambió la forma, por decirlo así, de como yo veía a los pacientes con Nefrolitiasis desde ese día”* (Healthcare professional, Chile)  *“(…) nos permite* [consultorías virtuales], *favorecer también la comunicación, como le decía yo, con el especialista, porque también es difícil, (…) y como unificar criterios, porque por más protocolos que existan uno igual tiene, genera dudas, y esas son las formas de aclararlo”* (Healthcare professional, Chile)  *“(…) la mayoría de la información es internacional quizás de Norteamérica o de Europa y uno dice “¿cómo hago para aplicar esas intervenciones, para llegar a un diagnóstico o un adecuado tratamiento en mi país, en Bogotá, en Fontibón?. ¿Qué hago si me están diciendo que tengo que hacer una arteriografía de miembros inferiores a todos los pacientes con pie diabético, por ejemplo?”. En las sesiones después de revisar todo eso a veces nos dábamos cuenta que si bien necesitábamos en algún momento de la atención llegar allá, pues el médico general no necesitaba pedir un doppler, por ejemplo, (…) porque precisamente podríamos llegar al diagnóstico sin pruebas diagnósticas que quizás sean costosas (…)”* (PP, Colombia)  **- Contributed to better control of chronic patients**  *“al final se beneficiaron nuestros pacientes, y los vimos como resultado al hacerle los controles, al mirar cómo estaba su patología. Vimos la mejoría en esos pacientes, los mantuvimos estables, les disminuimos las intervenciones a nivel de urgencias por descompensación”* (PP, Colombia)   - 1. **Improved appropriateness of referrals to secondary care**   - **Contributed to reducing unnecessary referrals to secondary care**  *“Houve um resultado concreto que foi a diminuição da fila de pacientes para endocrinologia e, uma das coisas que me foi colocado como a causa dessa diminuição foi a redução da necessidade de mandar o paciente para o endocrinologista, a Atenção Básica dando conta de conduzir esse paciente”* (Healthcare manager, Brazil)  *“Si porque además íbamos... el médico que está haciendo el triaje de las interconsultas, él participa de las videoconferencias. Entonces de alguna manera, él es un insumo, digamos, la videoconferencia, para después revisar la interconsulta, y ver qué corresponde y qué no corresponde, y él devuelve esas interconsulta a los médicos para que los haga de acuerdo al protocolo que está descrito. Entonces yo creo que sí nos sirve”* (Healthcare professional, Chile)  *“Entonces se aclaró un protocolo del manejo: en estos casos se remiten, en estos ustedes pueden continuar o en nosotros se los contra remitimos y ustedes los continúan manejando. Eso mejoró bastante, sobre todo el programa de crónicos en el que yo estuve participando. Más que fue el de XXX* [Hospital]*, ahí se dio bastante mejoría en este aspecto”* (PP, Colombia)  **- Contributed to improving early detection and timely referral of patients with complications**  *“Considero que si ha habido algunos cambios sobre todo en el primer nivel en detectar oportunamente a embarazadas de alto riesgo y canalizarlas en el mero momento. Antes era un poquito más tardado el envío”* (Healthcare professional, Mexico)   - 1. **Improved administrative coordination of patient access across care levels**   **- Fostered direct communication to facilitate access to SC and cross-level monitoring**  *“(…) son pequeñas cosas que van cambiando y que uno no las va percibiendo realmente, pero el hecho ya que uno pueda, eh..., preguntar por un usuario tal, hacerle el seguimiento. Por ejemplo hay usuarios que los mandamos con interconsulta al hospital, sin intervención aún porque le correspondía al hospital, pero después uno puede llamar para..., o poder preguntar por correo pa' ver cómo sigue el usuario, o si es que o el usuario es derivado para acá (…)”* (Healthcare professional, Chile)  **- Contributed to faster access for patients on waiting lists**  *“(…) en atención primaria como vivimos más cerca de la gente (…) recibimos el reclamo habitual: "Ay, pero tantos años que estoy esperando, ¿pero cómo?", y no se soluciona, y mi papá tiene este problema y mi hijo tiene este otro, etc.", y uno empezaba como a buscar cómo hacerlo, a mandar un ordinario, a mandar un correo electrónico, a buscar con quién me comunico para buscar soluciones. Ahora es como fluido…, hoy día!, (…) me llaman por teléfono, del hospital para darme una información para un usuario por el que yo pregunté ayer (…) y hoy día me llaman por teléfono para decirme: ah mire lo que hicimos fue esto, vamos a hacer esto otro, y dando la información”* (Healthcare manager, Chile)  **- Contributed to a reduction in administratively inadequate referrals**  *“(…) la recepción que hubo por parte de los especialistas. A mí me tocó con cardiólogo ¿ya?, y se dio el tiempo, digamos, de explicarnos los problemas que ellos tienen con algún tipo de interconsulta, ¿ya?. Entonces esas cosas que él nos dijo nosotros también las tratamos de ver de qué manera las podemos ir puliendo un poco, para que vayan un poco más completas o que sean más atingentes a los que ellos puedan resolver”* (Healthcare professional, Chile)  *“Para mí es útil, porque (…) entonces puedo parar interconsultas y puedo hacerle preguntas al médico: "doctor pasa esto, a la interconsulta le falta tal dato", y podemos coordinar para que la interconsulta no se vaya y quede como no pertinente y quede se vaya pertinente al tiro desde acá”* (Administrative professional, Chile)  *“(…) que los funcionarios sean conscientes de que a veces entregar una información errónea significa para el usuario un gasto de recursos innecesarios. Entonces en sentido esta estrategia potencia el buen convivir, o el buen tránsito más bien dicho, del usuario por la red de salud, porque los equipos se conocen con su contraparte”* (Healthcare manager, Chile)  **- Contributed to providing the patient with more adequate information on access to the other level**  *“Los médicos, ponte de atención primaria, cuando fueron allá y vieron el lugar que va el usuario, cómo tiene que conectarse, en dónde tiene que entrar y vieron los pequeños detalles (…) así como el detallito más básico, de donde entrega el papel, de donde tiene que ir (…). Entonces cuando después le transmiten al equipo, hoy día le transmiten con conocimiento de lo que tiene que pasar al paciente. O sea, “usted va a ir a tal lado” es distinto que cuando lo dices como autómata y no tienes idea en realidad, porque los libritos, los instructivos nunca logran la foto de la realidad”* (LSC, Chile)  *“Uno tiene más consciencia y uno le explica a los usuarios, “que es lo que me toca a mí”. Vienen a preguntar por su interconsulta porqué fue derivado al hospital, yo les explico: “miren lo que pasa es que el hospital se va a demorar en llamarlo porque tienen mucha lista de espera”” (*Administrative professional, Chile*)*   - 1. **Impact limited to the specialities that participated, or little impact on coordination**   *“no hubo participación de todos los especialistas con los que uno trabajaba. Entonces, si nosotros trabajamos con el doctor X, él entendía lo que yo hacía en mi consulta, porque había una coordinación entre los dos, pero los especialistas del Hospital XXX no entendían lo que el médico general hacía porque no estaban enterados de la dinámica. (…) Teníamos algunos choques con otros internistas de aquí, porque uno iniciaba algún tratamiento que aprendimos dentro del proceso y ellos decían: “¿pero por qué le hicieron esto?”. Entonces hubo choques, nos cambiaban los tratamientos porque no entendían la dinámica”* (PP, Colombia)  *“No creo que se haya logrado la mejoría de la comunicación (…) porque lamentablemente la presencia de los especialistas fue muy reducida. Entonces tampoco cumplió el objetivo en ese sentido”* (PP, Colombia)  *“De la plataforma* [sistema de comunicación on-line] *imaginándome la situación que ya todos conocemos de los centros de salud, los tiempos, los formatos y todo (...) tendrían que emplear cierto tiempo para meterse al sistema, llenar lo que pide el sistema, enviar la pregunta, y eso yo creo que significó el hecho de que no la utilizaran”* (LSC, Mexico)  *“El problema es ese que no logramos impactar* [con el sistema de comunicación on-line]*, no impactamos, al menos yo siento que no logré hacer lo que yo quería con el grupo, o sea de motivarlos”* (PP, Mexico)  *“En el sistema de comunicación en línea quizá no se tuvo el cambio que esperaban, no funcionó, tuvo algunas fallas, bueno por lo menos ya vimos que eso no funcionaba”* (PP, Mexico)  *“El resultado final realmente…. Es un pequeño avance, pero no todo lo que yo hubieses deseado que fuera”* (LSC, Uruguay)  *“En definitiva ha sido tan poco el movimiento que no podés evaluar si ha surgido algún tipo de cambio en la coordinación. Me parece que los números no te dan”* (LSC, Uruguay)  *“Yo creo que sí, que por lo menos ha sido un puntapié inicial de parte del primer nivel de mejorar lo que es la referencia, porque saben que están siendo evaluados esos formularios”* (PP, Uruguay)  *“No, ni mejoró, ni empeoró, ni nada, un papel más que molesta”* (Healthcare professional, Uruguay) |

| **Table S2: Examples of the category ‘Contribution to improving factors that influence coordination across care levels’** |
| --- |
| **2.1. Improvement in interrelationship factors between health professionals in the network**  **- Helped to get to know professionals of the other level and their work context and fostered personal connections**  *“(…), esa videoconferencia que se hizo, fue la primera como acercamiento que tuve más a APS. Mañana tengo otra (…), entonces igual tenemos ya una mayor relación, conozco más los nombre, y también se han hecho Whatsapp de cada comuna. Entonces de repente cuando vienen consultas o casos clínicos, me mandan un mail o me escriben por Whatsapp”* (Healthcare professional, Chile)  *“Sí, porque muchas veces me doy cuenta que..., de que ha pasado es que los especialistas no tienen idea de las cosas que están y no están disponibles en APS. Como nosotros también muchas veces no sabemos qué está y que no está disponible en el hospital”* (Healthcare professional, Chile)  *“Yo creo que son súper importantes ya que genera como este vínculo que te comentaba anteriormente...”* (Healthcare professional, Chile)  *“La idea* [de las capacitaciones] *era como cubrir todos los lados o las caras de la moneda, el acercamiento de persona a persona, de médico a médico, el verse las caras, el saber a dónde voy a mandar, el saber cómo voy a mandar, como voy a referir”* (LSC, Mexico)  **- Improved opinion of the other care level**  *“En relación a la inducción, creo que ha sido súper enriquecedor para las personas que han vivido el proceso, a (…) en términos de que es bueno conocer lo que el otro hace. Porque yo me hago una imagen teórica de lo que el otro hace, hay a veces, muchas veces, prejuicios en base a eso. Así que cuando lo conozco, mido y veo toda la carga asistencial que tiene, o el espectro de posibilidades que pueden tener. Digamos, es bastante bueno para comprender por qué no, o sí, se hicieron ciertas acciones”* (Healthcare professional, Chile)  *“(…) este estudio (…) como que ayudó como a refrescar un poco la visión de: "oh, en realidad igual no somos tan malos". Así como que hasta lo he escuchado de equipos: "no, en realidad nos conocemos, mira quizás hablamos solo por correo y tú en realidad..., no era como tu mala intención a no querer recibirme, no tenís recursos, digamos, ¿cómo lo hacemos?", y eso yo creo que puede aportar harto el Equity”* (Healthcare professional, Chile)  *“entonces a medida que van sucediendo las sesiones, creo que me beneficio bastante, y no solo eso sino que también se sensibiliza sobre la dificultades que tiene un médico general, y que uno ha olvidado porque ya está en otro momento de la atención de los pacientes. Ellos tienen que enfrentarse a mil cosas”* (PP, Colombia)  *“Sí, se fomentó esta cercanía entre los actores que estamos aquí involucrados. Pues ya no los vemos tan lejanos, ya no los vemos como los integrantes del equipo contrario, sino que ya los vemos como integrantes del mismo equipo”* (PP, Mexico)  **- Improved trust, willingness to collaborate, communication between participants**  *“[...] ter um momento que se conheça, que olhe nos olhos e faça com que, quando você ligar, quando tiver utilizando alguma ferramenta, saiba com quem está falando [...] A gente sente nos relatos dos profissionais a importância desse momento, de sair cada um do seu espaço [...] falando no telefone, mas eu estou sabendo quem está do outro lado”* (Healthcare professional, Brazil).  *“las video* [conferencias virtuales] *nos han servido también para establecer lazos entre las enfermeras de allá que están encargadas de otras áreas y las enfermeras nuestras (…) Se pasan los Whatsapp y todo, y también hemos acelerado, digamos, la..., la ¿cómo se llama?, la posibilidad de resolver cosas. Porque tampoco se conocían, las enfermeras de cirugía allá con las enfermeras que hacen las curaciones acá, las que ven pie diabético allá con las que ven pie diabético acá. Entonces quedan como: "oh, yo pensaba que ustedes…", "oh, yo creía que ustedes…". No había el conocimiento real que hacía el otro”* (Healthcare manager, Chile)  *“(…) es súper bueno generar esos lazos, porque a mí, por ejemplo, me llegan muchos pacientes esperando desde el año 2014 y yo acá, y digo: “chuta ¿y a quién llamo?, ¿y cómo consigo?” Y llamo al contacto que tengo de allá* [en el hospital] *pero resulta que no me contesta, (…) O muchas veces yo me meto incluso a computadores ahí, a buscar ahí los número de teléfono porque no hay como un contacto tan directo con las otras especialidades. Entonces ahí yo creo que es como importante quizás repetirlo* [visitas entre niveles] *para ir abarcando más especialidades”* (Administrative professional, Chile)  *“Han sido estrategias que (…) han producido un cambio en términos de que... yo creo que la red hoy día se ve, estamos, hay un nivel más colaborativo entre los niveles de atención. Yo siento que hay una disposición más colaborativa”* (Healthcare professional, Chile)  *“(…) al sentirse de sitios diferentes, como que no querían unir sus ideas y conocimientos, pero ya al sentirse uno sólo como parte de un grupo, pues comenzaron a generar identidad …Al sentirse uno solo, al pertenecer a un grupo, como que cambió la parte mental, como que si todos vamos a estar juntos, todos vamos a participar y todos vamos hablar”* (PP, Colombia).  *“Hay veces que yo voy al hospital y busco alguno de los que ya conozco* [participantes del sistema o sesiones de capacitación] *y me han ayudado mucho* [a acordar envíos de ginecología](…) *No los conocía*, *muchos de los doctores que trabajan en el* [centro de atención especializada] *han sido porque hemos ido a cursos* *y ahí nos vamos conociendo”* (Healthcare professional, Mexico)  **- The PAR process improved interrelationship factors between managers of the network**  *“Yo creo que había más desesperanza (…) Lo único que se nos ocurría era llamar al director y decirle..., o reclamar y decir..., la onda como del reclamo individual, pero sin mucha confianza en los equipos. Yo creo que lo que hay es que existen otros equipos, que aunque no esté el director, a lo mejor con esos equipos también se puede construir. Hay más canales. Sí, más canales y la sensación de red. O sea, como de que hay otros con los que también se puede estirar la mano y decir: "oye, ¿ por qué no hacemos esto?"”* (Healthcare manager, Chile)  *“El Comité conductor* [LSC]*, digamos que genera un espacio que permite mantener cierta continuidad, que probablemente en otros momentos, sin el proyecto, la desarticulación hubiese sido mayor y la fragmentación aún más”* (Healthcare manager, Chile)  *“Pero no es la desesperanza que había antes, porque hace 4 o 5 años si tu hubieras preguntado, era una situación como, era una situación de estar en un descampado, una cuestión como de estar un páramo, una sensación como de estar sólo. Pero ahora se siente como de que puedes hablar con otros, uno tiene cómo resolver alguna cuestión”* (LSC, Chile)  *“llegamos todos con las defensas aquí… y fuimos dándonos cuenta que en realidad nos fuimos comunicando bien, nos fuimos escuchando, y entendiendo desde la empatía absoluta (…) Pudimos entonces ir construyendo (…) Por eso es que es tan bonito lo que hemos hecho, porque todos hemos ido aportando un ladrillito para que esto funcione”* (LSC, Chile)  **2.2. Improved training/updating of PC and SC doctors**  *“Encuentro que absolutamente útil, absolutamente útil, es educación médica continua. O sea en el fondo tienes que buscar la forma de mantener a las personas actualizadas, y también sirve incluso para los que preparan acá y tienen que hacer revisiones. Una educación médica continua también para el que tiene que estar preparando la videoconferencia”* (Healthcare professional, Chile)  *“estas capacitaciones, para mi fueron muy importantes por eso, porque nos permitieron actualizarnos, no permitieron estar en contacto con otros profesionales, con otros médicos y nos hicieron entender que trabajar en equipo es muchísimo mejor”* (PP, Colombia)  *“el más beneficiado desde la visión desde lo que yo estoy haciendo, soy yo porque precisamente estoy haciendo una actualización de diferentes temas. Al orientar los casos, quien más aprende soy yo”* (PP, Colombia)  **- The PC doctors who participated became sources of reference in their units**  *“(…) y eso yo encuentro que es súper, súper, súper valioso, súper valioso, porque en el fondo no es solamente lo que uno está diciendo con sus conocimientos y su formación de médico recién egresado, sino que es algo que es una percepción o una educación que nosotros recibimos, que está avalada por un especialista que hizo esta reunión en este tema. Por lo tanto encuentro que es súper valioso porque eso te da autoridad frente a los colegas, y puedes en el fondo conversarlo (…)”* (Healthcare professional, Chile)  **- Contributed to improving PC doctors’ management of patients and the appropriateness of referrals to SC**  *“en el manejo, se mejoró el tratamiento, nosotros aprendimos a identificar riesgos, a identificar problemas y a prevenirlos en las patologías crónicas que manejamos. Eso lo aprendimos de ellos, de los especialistas. Entonces ya el protocolo de manejo con respecto a una enfermedad, deja de ser exclusividad del especialista. Pasa a ser también propiedad del médico general y eso redunda en una mayor calidad de la atención al paciente”* (PP, Colombia)  *“Uno ya comienza a ver el impacto entre los médico. Los médicos ya comienzan a dar tratamientos y manejos conforme a la estandarización que se han dado en estas reuniones. ¡Miren!, de las reuniones han salido buenos productos”* (LSC, Colombia)  *“Con el desarrollo de esta reunión nos dimos cuenta que los médicos generales a veces remitíamos pacientes a quienes podíamos manejar, y eso lo aprendíamos de los especialistas”* (PP, Colombia)  *“Sí sirvió, las sesiones académicas entre especialistas y nosotros y bueno pues sí, “esto no lo puedo mandar por esto, esto y esto, esto lo mando con esto, esto y esto”. A veces había diferencia de opinión, bueno, según la norma dice que se lo mande si tiene tales o cuales cosas, y en hospital dicen “no, no, no, si no tiene esto ni me lo mandes o ¿para qué me lo mandas?””* (PP, Colombia)  *“Algo muy sencillo es que otro de los hospitales receptores notaron que en un envío la mujer iba con la documentación adecuada, con el pinzamiento, por ejemplo, de sangrado adecuado, y entonces era lo que ya habían aprendido en el curso”* (LSC, Mexico)  **2.3. The PAR process fostered professionals’ interest in participating in the interventions**  *“*[la metodología] *excelente diría yo, porque pues era como una necesidad sentida de los médicos generales tener una retroalimentación, un diálogo, una comunicación más directa con los especialistas”* (PP, Colombia)  *“(..) o projeto de intervenção foi construído com a gente, levando em conta a realidade local, então eu acho que o legal foi isso, essa questão de adequar mesmo a sua necessidade”* (Healthcare manager, Brazil) |

| **Table S3: Examples of the category ‘Opinions and conditions for the sustainability of the interventions’** |
| --- |
| **3.1. Opinions and conditions for the sustainability of the interventions**  **- Desire for the implementation of the interventions to continue**  *“Parte es de los que hacemos las sesiones (médicos generales y especialistas) (…) solo necesitamos el espacio y obviamente si la red apoya, pues esto no tendría por qué cambiar, porque es que así como estamos 2, 3 4 años y ojalá mucho tiempo”* (PP, Colombia)  *“Yo creo que hay que seguir manteniéndola activa, digo activa como viva, por decirlo de alguna manera. Yo creo que lo que hay que seguir es como estimulando a los especialistas y sumando a otros”* (Healthcare professional, Uruguay)  **- Institutionalization of virtual consultations**  *“Facilitarlo, yo creo que las intervenciones como tal, creo que han llegado a un grado de institucionalización que no creo que se pierdan, al menos las de las consultorías virtuales”* (Healthcare managers, Chile)  *“(…) yo creo que eso quedó en la orientación programática ministerial. O sea ahí el comité del Proyecto logró instalarlo a un nivel que yo creo que no se desarticula”* (Healthcare manager, Chile)  *“Sí, y ahí ocurre un hito importante que tiene que ver con que se incorpora (…) a la programación. (…) y podemos incluso programarla para el próximo año y asegurar que estén las personas que corresponden para cada una de sus presentaciones. Me parece ese también un hito bien importante (...) en la videoconferencia”* (LSC, Chile)  - **Facilitators of the sustainability of the joint meetings: alignment with/incorporation into state policy, favourable results and professionals’ interest in the interventions**  *“Podemos tomar esta estrategia y decir, a través de esta estrategia podemos implementar las rutas de atención integral en salud”* (PP, Colombia)  *“Creo que tenemos dos cosas a favor* [para la sostenibilidad]*, en el plano de capacitación de salud materna con redes integradas, tenemos el plan de atención a la salud materna, el plan estatal que ya fue firmado por COESA, ya tenemos primero ese documento normativo (…)”* (LSC, Mexico)  *“*(…) *yo creo que como estamos en un mundo en que el dinero es lo más importante, yo creo que si esto es una prestación valorada. Para el hospital va a ser muy importante crearla y mantenerla, ¿ya? (...)desde el punto de vista asistencial es solo ganancia, ya que ese paciente se ahorre $1500 en micro de venir para acá, y que esté parado en una fila no sé cuánto, y que su médico le dé la solución en su consultorio ya es maravilloso. No le veo ningún contra (…) sería como muy tirado de las mechas como dicen aquí que alguien se oponga a una iniciativa tan buena”*  (Healthcare professional, Chile)  *“Es una estrategia que ya se conoce, que ha dado buenos resultados, simplemente es continuar un proceso que ya fue iniciado y en el que muchas personas ya están involucradas”* (PP, Colombia)  *“Sería una tristeza que esto llegara a desaparecer, siendo tan efectivo*” (PP, Mexico)  *“(…) de la sostenibilidad agregaría también que la institucionalización tiene que realizarse, tiene que darse (...) es motivo de satisfacción y de regocijo pues los logros que se tienen”* (LSC, Mexico)  *“Si hay un compromiso del subgerente de servicios de salud, ya ese se desarrolla solito. El análisis de casos se desarrolla solito con los médicos, porque ya ellos se van a reconocer como los vínculos (…) en cada una de las unidades, y van a liderar ese proceso, ya lo van a sentir suyo. Cuando las personas lo sienten suyo, sienten esa, eso es trabajo en red”* (Healthcare manager, Colombia)  **3.2. Threats or difficulties for the sustainability of the joint meetings**  **- Lack of management’s support for their maintenance**  *“Un poco de temor porque, en realidad, en esta horizontalidad* [método IAP]*, porque finalmente, esta re... la responsabilidad de la permanencia igual la ha tomado el equipo investigador, entonces es como, tiene que aflorar, dentro de esa horizontalidad, igual un liderazgo que permita la permanencia, entonces si no se identifica ese liderazgo, o no surge espontáneamente, puede ocurrir que se...”* (Healthcare manager, Chile)  *“La sostenibilidad a mí me parece un poco amenazada si es que el Servicio de Salud no se pone un poco..., en el sentido de avanzar y generar una práctica habitual con todos los centros de salud, con todas las comunas, digamos.”* (Healthcare manager, Chile)  **- Change of government**  *“Hay incentivos para que no ocurra, porque en el fondo en un contexto de cambios políticos, que genera mucha incertidumbre, debiera aflorar que este espacio es relevante, que permite, no sé si defender, pero hacer prevalecer elementos que se han ido consensuando más allá de las miradas políticas, partidistas me refiero, con una visión más sanitaria y de sentido de red que debiera trascender, pero me da temor, me da temor que al no estar asegurado los recursos… O sea si partimos desde el gestor de red, que uno podría decir: "oye aquí hay una institucionalización mayor, por ejemplo, del espacio", no estoy tan segura de que pueda sobrevivir. ¿Sí?”* (Healthcare manager, Chile)  *“(…) nosotros tenemos una importante preocupación con el tema del cambio de gobierno, (…) porque particularmente no sabemos con qué recursos adicionales vayamos a contar o no y a lo mejor vamos a tener restricciones. Entonces, cómo asegurar dejar a nuestro equipo con buenas condiciones, funcionando. O sea que en el fondo iniciativas como esta no se caigan porque faltó no sé, la cámara, porque se cayó no sé qué o el sistema no funcionó”* (LSC, Chile)  **3.3. Necessary elements for the sustainability of the interventions**  **- Institutionalization of the interventions**  *“... se isso (a intervenção) for institucionalizado[...]então é mais fácil!”* (Healthcare professionals, Brazil)  *“Y(…) sobre la cuestión de la sostenibilidad agregaría también que la institucionalización tiene que realizarse, tiene que darse (...)”* (LSC, Mexico)  **- Institutional support**  *“Para que esto se sostenga, básicamente es una cuestión de voluntades (...) y también de que las redes se hagan cargo y se responsabilicen de la integración ¿ya?. Yo creo que en lo particular se trata de implementar un modelo de redes integradas. Pero en la práctica (...)si existe un liderazgo, existe un interés en querer hacerlo, yo creo que el éxito es bastante garantizado en ese sentido ¿ya?”* (Healthcare manager, Chile)  *“Todo depende del compromiso de la alta dirección, en nuestro sistema de salud y más en un ambiente público donde la producción de cada uno de los profesionales es lo que hace que haya una sostenibilidad financiera. Este tipo de situaciones a veces desde las direcciones no es tan oportuna y no permiten garantizar que una estrategia como ésta se logre mantener en el largo plazo”* (LSC, Colombia)  *“Primero era como anclar todo este potencial que tenía el seminario a un compromiso institucional”* (LSC, Mexico)  **3.4. Strategies for the sustainability of the interventions**  **- Incorporation of the interventions into network planning/local policy**  *“Se for vista...numa linha longitudinal [...] não centralizada em profissionais, mas entendida enquanto política do município [...]. Vai ajudar para resistir* [a intervenção]*”*( Healthcare manager, Brazil)  *“(…) la visita inter-niveles quedaron así como chispazos, digamos, no están todavía insertas dentro de un proceso (…) Debiesen ser parte de un proceso de inducción de la red”* (Manager, Chile)  *“(…) Yo creo que en ese sentido nuestra red de salud está en pie como para poder organizar a través de sus referentes, sin la intervención de la Universidad, una visita de la inducción (…). Y de cómo se van incorporando estos elementos al quehacer diario o a la planificación en red. En ese sentido yo creo que eso es súper importante para la sostenibilidad ¿ya?. Y eso claramente va de la mano con poder abordar desde el punto de vista de institucionalizar estas intervenciones”* (Healthcare manager, Chile)  *“Esperaríamos que el Plan Veracruzano 2017-2018 corresponda a un Plan Veracruzano 2018-2024 para poder dar línea seguida a lo que se está haciendo capacitaciones”* (LSC, Mexico)  **- Allocation of resources**  *“..., incluso que hubiera una oficina donde se haga esto, porque siempre estamos pidiendo la sala de dirección. Pero puede haber "x" necesidad, y nos quedamos sin salita. Entonces el hospital debería tener, no sé po, una sala como esta con su equipo instalado en que no se movieran y que ahí se hagan las teleconferencias, la... como quieras llamarla”* (Healthcare professional, Chile)  *“Tenemos que definir tiempos específicos. Eso sería lo ideal, definir los profesionales, los temas, no solamente crónicos, si no, definir los perfiles y los tiempos”* (Healthcare manager, Colombia)  *“El 90 % de la población contratada está por OPS, el otro 10% es de planta. Si se logra incluir dentro de los contratos por OPS el cumplimiento de las horas para este tipo de espacios académicos, aunque sea poco tiempo desde lo contractual, que se garantice el espacio para ese 90 %, porque el 10% de planta ya lo tiene y le toca ir”* (LSC, Colombia)  **- Appointing staff to take charge and defining their roles in the implementation process**  *“Si designaran a una persona cabeza responsable de querer continuarlas, habría la continuidad”* (PP, Colombia)  *“(…) buscar esta institucionalización, quizá desde el propio organigrama, en alguna parte que pudiera ser incluida la subdirección (...). Eso de tener la disponibilidad de tener un área que se encargue de la capacitación”* (LSC, Mexico)  **- Making processes and spaces official**  *“Estar en eso, y formalizarlo cuando corresponda o llevarlo a la instancia que tenemos instaladas como las instancias formales para la toma de decisiones. Que trascienden a toda la red asistencia. Entonces esa es una aprensión que tengo respecto a esta instancia, que conociendo a nuestros hospitales, que son muy pro, pero que de repente se les olvida que no es la relación con un hospital con una CESFAM, o con una comuna, etc., etc., sino que aquí se producen relaciones súper transversales, y más complejas que eso, es donde empiezan de repente a quedar hilos afuera y después no..., no..., los resultados no son tan buenos”* (Healthcare manager, Chile)  **- Raising awareness of the usefulness of the intervention through disseminating its results and evaluating its cost-effectiveness**  *“creería que una de las cosas es la sensibilización a los profesionales administrativos de que la capacitación de fortalecimiento de competencias, les va a mejorar la calidad en la prestación de sus servicios y les va a mejorar de alguna u otra forma. Y no estoy hablando de manera indirecta, sino de manera directa su producción”* (LSC, Colombia).  *“La sensibilización inicialmente con la dirección. Y es que la alta dirección no es solamente la gerente, es el comité, también el nivel táctico. Y el nivel táctico, somos nosotros los líderes de centro e inclusive táctico y operativo. Porque si bien es cierto, yo dirijo y administro, yo también tengo que ponerme la camiseta y decirles “venga esto les va servir por este y este motivo, vayan”. O sea ayudo, soy partícipe de esa sensibilización pero yo como líder, primero tengo que convencerme, eso en muchos de nosotros no está, esa sería también otra propuesta”* (LSC, Colombia)  *“Si yo mejoro en mi práctica clínica diaria y también estímulo a mis compañeros a que hagan lo mismo en su unidad, probablemente eso se va a ir extendiendo hasta el punto que cause impacto, y que digan la manera de desarrollar la estrategia de esta plataforma tiene sus ventajas. Deberíamos apoyarla más”* (Healthcare professional, Colombia)  *“Demostrar con... en forma objetiva lo eficiente que ha sido. Y tampoco se le puede pedir un mes, pero después de un año ver los resultados y, y, y bueno, frente a las cosas objetivamente demostradas que son buenas”* (Healthcare professional, Chile)  *“deberían demostrar de alguna manera, divulgación algún impacto sobre el paciente algún benéfico en cuestión de complicaciones de menos hospitalizaciones, algún parámetro”* (PP, Colombia) |

| **Table S4: Examples of the category ‘Opinions and conditions for the applicability of the interventions to other contexts’** |
| --- |
| **4.1. Opinions on the applicability of the interventions to other contexts**  **- Interventions are applicable, their application is recommended**  *“É perfeitamente reprodutível, a gente espera que consiga reproduzir para todas as linhas de cuidado”* (Healthcare manager, Brazil).  “*Yo siento que es necesario, porque es que resulta que las subredes tienden a desaparecer, resulta que el modelo debería a futuro volver eso una sola red. Entonces allá también hay que hacerlo de manera que sea beneficio no solo al paciente que atienden en esas localidades (…) debería extenderse a las otras redes.*” (Healthcare manager, Colombia)  - **They contribute to tackling a common problem in different settings**  *“— Sí, yo creo que sería útil, sería útil en otros centros, yo en lo personal trabajé en el XXXX, y me doy cuenta de que muchas veces se cometían hartos errores al derivar, o a hospitalización domiciliaria o a control en APS para tal cosa que no estaba disponible obviamente, (…) . Entonces es súper bueno que los médicos que trabajan en el hospital sepan qué está disponible afuera y los médicos que trabajan afuera estén súper conscientes de lo que está disponible adentro del hospital, como para saber qué hacer”* (Healthcare professional, Chile)  “*son absolutamente replicables, porque el problema común es medio sistémico, no lo vivimos solo este servicio de salud”* (LSC, Chile)  *“Es evidente que aquí en la red ha tenido un éxito bastante importante. Las otras redes desde el punto de vista teórico tienen que tener los mismos procesos o muy parecidos. En este caso entre médicos generales y especialistas, en realidad no tendría dificultades, se podría implementar”* (PP, Colombia)  **- They are aligned with/incorporated into local or national network policy**  *“La implementación del modelo de atención en redes de servicios integrales de salud, en lo que está empeñada esta administración del distrito, digamos que coincide muchísimo con la intención de esta estrategia Equity, porque de alguna manera es un trabajo en redes, y hacia el futuro, esta estrategia está planteada dentro de la implementación de un modelo de atención en redes”* (Healthcare manager, Colombia)  **- Benefits of replicating the PAR method**  *“A mí me parece altamente replicable, siempre y cuando exista algún proceso previo de diagnóstico y exista un grupo que quiera trabajar en mejorar los problemas que afectan… O sea yo creo que acá todos concordamos que el diagnóstico que había acá es que ésta era una red muy fragmentada, donde no nos conocíamos mucho, donde había dificultades para trabajar. Y eso al día es fundamentalmente distinto. Por lo tanto mientras exista un grupo que quiera superar esas barreras yo creo que es altamente replicable”* (LSC, Chile)  **4.2. Necessary elements to apply the interventions in other contexts**  **- Interest of professionals and managers in improving care integration**  *“Para que esto se sostenga, básicamente es una cuestión de voluntades, y para replicarlo yo creo que también aplica un poco lo mismo, de tener la voluntad de... y también de que las redes se hagan cargo y se responsabilicen de la integración ¿ya?”* (Healthcare manager, Chile)  *“El tema de la voluntad política, del interés, de la disposición de los directores científicos, pero también de los directores de las redes en poder implementar la estrategia”* (LSC, Colombia)  *“esta estrategia se puede desarrollar, claro, se puede replicar, todo depende de la disposición de las personas y de la parte administrativa, pero tiene todo el modelo para replicarse en otras redes”* (PP, Colombia)  **- Presence of leadership in the network with conviction and perseverance in the implementation process**  *“es necesario ser constante (…) es súper importante considerarlo, por si alguien, o "x" quisiera replicar esto, que es importante eso, la constancia.”* (Healthcare manager, Chile)  *“Yo creo que el tema es como del impulso y del sostén durante un tiempo. Tener personas ocupadas de que esto dinámicamente funcione, como un acompañamiento, con gente que esté dedicada”* (Other professionals, Uruguay)  **- Institutional support in the allocation of resources needed for implementation**  *“Lo primero es que desde el inicio sea con cronograma de actividades previo, para que tanto se puedan agendar las horas médicas con tiempo. Como que el médico tenga un tiempo determinado para poder presentar los casos que más le complican”* (Health professional, Chile)  *“Necesitas el apoyo del director de la institución, (…), a nivel nacional, digo. Tener apoyo a nivel político, de normativas, no sólo con la voluntad…”* (Administrative professional, Uruguay)  **- Improve the design and implementation of offline virtual consultations and referral forms**  *“Por desgracia, sino hay chicotazo la gente no trabaja, pero si implementas un programa bien implementado pero sobre todo con exigencias (…) Porque te estoy exigiendo a que me lo hagas, pero si yo te dejo a ti manga ancha a que lo hagas, baja mucho la cosa”* (PP, Mexico)  “*Buscar cómo se les puede exigir que completen esto, o capacitarlos de cómo hacer una buena referencia”* (PP, Uruguay)  *“Se discutió mucho sumarle la figura de enlace, creo que son cosas a considerar a futuro que podrían mejorar aún más la coordinación entre los niveles de atención”* (LSC, Uruguay)  *“Hacerlo más participativo con los médicos, con algún tipo de devolución o informe para ellos”* (Administrative professional, Uruguay) |
