# Supplementary material for: Can care coordination across levels be improved through the implementation of participatory action research interventions? Outcomes and conditions for sustaining changes in five Latin American countries
Source: BMC Health Serv Res. 2020 Oct 12;20:941. doi: 10.1186/s12913-020-05781-7 (PMC7552474; doi:10.1186/s12913-020-05781-7)
Supplement: Supplementary file 2 — Additional file 2. Table 1. Characteristics of the implemented interventions in the study networks. Description of data: Description of the characteristics of the implemented interventions. [file 12913_2020_5781_MOESM2_ESM.docx]

**Table 1. Characteristics of the implemented interventions in the study networks**

| **Type of interventions** | **Brazil** | **Chile** | **Colombia** | **Mexico** | **Uruguay** |
| --- | --- | --- | --- | --- | --- |
| **Joint meetings** | ***Joint discussions of clinical cases in mental health*** | ***Joint virtual clinical conferences*** | ***Joint meetings for discussion of clinical cases and medical training*** | ***Joint training sessions*** |  |
| **Characteristics** | Discussion of clinical cases (mental health), face-to-face | Online clinical conferences (discussion of clinical cases, referral criteria and follow-up) (any condition) | Discussion of clinical cases and medical training (chronic diseases), face-to-face | Training sessions based on clinical cases (maternal and perinatal care and chronic diseases) |  |
| **Type of participants** | PC teams and psychiatrists | PC and SC doctors and other professionals | PC and SC doctors and other healthcare professionals | PC and SC doctors |  |
| **Offline virtual consultation between PC and SC doctors** | **Virtual consultation between levels** |  |  | ***Virtual communication system between levels*** |  |
| **Characteristics** | Asynchronous virtual consultations in mental health via email |  |  | Asynchronous virtual consultations for chronic diseases and maternal and perinatal care via digital platform, and clinical protocols/guidelines repository |  |
| **Type of participants** | PC doctors and psychiatrists |  |  | PC and SC doctors |  |
| **Others** | ***Diabetes shared care guidelines*** | ***Induction program for working in network*** |  |  | ***Strategy to promote use of referral and reply letter*** |
| **Characteristics** | Creation and implementation of shared care guidelines (focused on essential practices and care pathways) | Cross-level bidirectional visits between PC and SC, information chart and audiovisual dossier on the network |  |  | Standardized format, flowchart and rules of use |
| **Type of participants** | PC doctors, endocrinologists and other professionals | Professionals of both care levels, but focusing on those starting to work in the healthcare network |  |  | PC and SC doctors and other professionals |

PC – Primary care; SC- Secondary care

More details on the characteristics of each intervention in: <http://www.equity-la.eu/en/publicaciones.php?t=PR>
