# Supplementary material for: Can care coordination across levels be improved through the implementation of participatory action research interventions? Outcomes and conditions for sustaining changes in five Latin American countries
Source: BMC Health Serv Res. 2020 Oct 12;20:941. doi: 10.1186/s12913-020-05781-7 (PMC7552474; doi:10.1186/s12913-020-05781-7)
Supplement: Supplementary file 3 — Additional file 3. Interview guide. Description of data: Topic guide for the analysis of contributions of the interventions to care coordination, necessary conditions and strategies for its sustainability and applicability to other contexts. [file 12913_2020_5781_MOESM3_ESM.doc]

**Additional file 3 - Interview guide on the evaluation of the implementation of interventions to improve care coordination taking a participatory action research approach (translated by the authors from the Spanish version) [[1]](#endnote-2)**

Date of interview:

Time of initiation/finalization:

Healthcare organization:

Location:

Collect the following data from each participant: name, age, gender, profession, position, institution, level of care and years working in the network (if applicable)

*The topics will be addressed in relation to the implementation of the intervention/s and their main components*

**PROCESS OF IMPLEMENTING THE INTERVENTION**

1. **What do you think of the process of designing and implementing *[name intervention]*? How did the intervention evolve over time? What changes were made? Why? What effects did they have?**

*If they are not mentioned, ask for opinions on:*

*- process of selection and design of the intervention*

*- planning the intervention*

*- dissemination / training for the intervention*

*- characteristics of the intervention, content, activities, resources, etc.*

*- monitoring / evaluation activities during the process*

*- role of key actors (Local Steering Committee (LSC), Professional Platform (PP), research team)*

1. **How was your participation in *[name the intervention]*?**

**FACTORS THAT HAVE INFLUENCED THE IMPLEMENTATION**

1. **What factors have facilitated the implementation/results of *[name intervention/ components]*? How did they facilitate it? Why?**

*If they are not mentioned, ask for opinions on:*

*- Healthcare system and health policies, changes in national and local governments, pressure from other actors*

*- Healthcare networks: institutional support, working conditions, pressure from other actors, resource allocation system, network objectives and strategies, healthcare model*

*- Professionals: interest in care coordination and intervention, implementation skills, attitude (disposition) or openness/resistance to change, confidence in the process of implementation*

1. **What factors have hindered the implementations/results of [name intervention/ components]? How? Why? Suggestions for improvements?**

*If they are not mentioned, ask for opinions on:*

*- Healthcare system and health policies, changes in national and local governments, pressure from other actors*

*- Healthcare networks: institutional support, working conditions, pressure from other actors, resource allocation system, network objectives and strategies, healthcare model*

*- Professionals: interest in care coordination and intervention, implementation skills, attitude (disposition or openness/resistance to change, confidence in the process of implementation*

**contribution of the implemented intervention**

1. **Did [*name intervention / components]* contributed to improving care coordination between levels of care? Which aspects? How? Why?**

*Explore opinions about the contributions of the intervention in relation to the defined objectives (for example, improving communication between PC and SC doctors, agreement on treatments, diagnostic tests, improving doctor’s knowledge and skills, improving referral/reply letter use, etc.)*

1. **Did [*name intervention / components]* trigger other changes in the network (in primary care centers / hospitals / etc.)? Which changes? How did the intervention trigger them? Why? What do you think of these changes? Have these changes contributed to improved coordination? How?**

*If they are not mentioned, ask for elements related to changes in policies, in the structure and organization of the healthcare network, in professionals, changes in other healthcare networks.*

1. **How did the participatory method contribute to the results of the intervention?**

**SUGGESTIONS FOR IMPROVEMENTS**

1. **What aspects of the intervention should be maintained/strengthened? Why? How?**
2. **What elements of the intervention should be modified? Why? How?**

**SUSTAINABILITY and APPLICABILITY**

1. **What do you think of the sustainability of the interventions over time in the healthcare network? What elements of the intervention facilitate its sustainability? Which other elements/strategies would be necessary?**
2. **What do you think about the implementation of this intervention in other networks? Which elements/strategies would be necessary to apply it?**

1. Vargas et al. Can care coordination across levels be improved through the implementation of participatory action research interventions? Outcomes and conditions for sustaining changes in five Latin American countries. BMC Health Serv Res. 2020 [↑](#endnote-ref-2)
